# Defect-free potassium manganese hexacyanoferrate cathode material for high-performance potassium-ion batteries

**DOI:** 10.1038/s41467-021-22499-0

**Published:** 2021-04-12

**Authors:** Leqing Deng, Jiale Qu, Xiaogang Niu, Juzhe Liu, Juan Zhang, Youran Hong, Meiying Feng, Jiangwei Wang, Miao Hu, Liang Zeng, Qianfan Zhang, Lin Guo, Yujie Zhu

**Affiliations:** 1 School of Chemistry, Beihang University, Beijing, P. R. China; 2 School of Physics, Beihang University, Beijing, P. R. China; 3 School of Materials Science and Engineering, Beihang University, Beijing, P. R. China; 4 Center of Electron Microscopy and State Key Laboratory of Silicon Materials, School of Materials Science and Engineering, Zhejiang University, Hangzhou, P. R. China; 5 CNOOC Research Institute of Refining and Petrochemicals, Beijing, P. R. China; 6 Key Laboratory for Green Chemical Technology of Ministry of Education, School of Chemical Engineering and Technology, Tianjin University, Tianjin, P. R. China; 7 Beijing Advanced Innovation Center for Biomedical Engineering, Beihang University, Beijing, P. R. China

**Keywords:** Batteries, Energy, Batteries, Batteries

## Abstract

Potassium-ion batteries (KIBs) are promising electrochemical energy storage systems because of their low cost and high energy density. However, practical exploitation of KIBs is hampered by the lack of high-performance cathode materials. Here we report a potassium manganese hexacyanoferrate (K_2_Mn[Fe(CN)_6_]) material, with a negligible content of defects and water, for efficient high-voltage K-ion storage. When tested in combination with a K metal anode, the K_2_Mn[Fe(CN)_6_]-based electrode enables a cell specific energy of 609.7 Wh kg^−1^ and 80% capacity retention after 7800 cycles. Moreover, a K-ion full-cell consisting of graphite and K_2_Mn[Fe(CN)_6_] as anode and cathode active materials, respectively, demonstrates a specific energy of 331.5 Wh kg^−1^, remarkable rate capability, and negligible capacity decay for 300 cycles. The remarkable electrochemical energy storage performances of the K_2_Mn[Fe(CN)_6_] material are attributed to its stable frameworks that benefit from the defect-free structure.

## Abstract (web summary)

Potassium-ion battery is a promising candidate for post-Li-ion energy storage but the lack of cathode materials hinders practical exploitation. Here the authors investigate defect-free potassium manganese hexacyanoferrate as cathode active material for high energy and long lifespan K-based cells.

## Introduction

Despite being widely utilized in electronics and electric vehicles, lithium-ion batteries (LIBs) are predicted to face the unsustainability due to the rarity and uneven distribution of lithium resources, making them difficult to meet the requirement of low levelized cost for the ever-increasing grid-scale energy storage applications^1^. For this reason, sodium-ion batteries (NIBs) and potassium-ion batteries (KIBs) have been considered as the promising complementary alternatives to LIBs owing to the utilization of earth-abundant and much cheaper sodium and potassium elements^2,3^. Besides, the electrode materials in NIBs and KIBs are usually free from toxic and expensive cobalt, which is one of the essential elements in state-of-the-art LIBs. Over the past decades, great progress has been made for non-aqueous NIBs which are being commercialized by few startup companies^4^.

Regarding the emerging KIBs, they not only share similar features with NIBs such as low-cost element as the charge carrier and the use of cheap and light aluminum instead of copper as the anode current collector, but also possess several additional unique advantages as follows. (1) The redox potential of K^+^/K is more negative than that of Na^+^/Na (and even Li^+^/Li) in some non-aqueous electrolytes, leading to potentially high voltages of KIBs that are similar to LIBs and relatively higher energy density than that of NIBs^5,6^; (2) the graphite anode can reversibly take and release K-ions with a theoretical capacity of 279 mAh g^−1^, making it possible to transfer the well-established graphite anode industry of LIBs to KIBs, whereas the anode for the commercialized NIBs utilizes the more expensive hard carbon^4^; (3) K-ion electrolytes reveal the highest ionic conductivity because of the weakest Lewis acidity and smallest Stokes radius of K-ions than those of Na-ions and Li-ions^7^. Although the aforementioned merits make KIBs very attractive, the success of KIBs, mainly relying on the cost effectiveness of KIBs over contemporary LIBs technology, can only be realized if ultra-long lifetime is achieved for KIBs, as recently emphasized by Yan and Obrovac through a detailed cost analysis^8^. The cycle life of KIBs mainly hinges on the charge–discharge reversibility of their electrode materials. On the anode side, reversible potassiation–depotassiation of commercial graphite anode with an impressive cycle life over 2000 cycles has been reported^9^. However, the reported cathode materials, particularly those with extractable K-ion source which can be paired with practical K-free anodes, usually exhibited unsatisfactory long-term cycling performance^5^. The lack of low-cost and high-performance K-containing cathode materials has hindered the development of practical KIBs.

Among the reported cathode candidates for KIBs, Prussian blue analogues (PBAs) have received tremendous attention^10–15^. The general composition of PBAs can be expressed as A_*x*_M[M′(CN)_6_]_1−*y*_□_*y*_•*n*H_2_O (0 ≤ *x* ≤ 2, *y* < 1), where A represents alkaline metal ions, M/M′ represent the transition metals (Fe, Mn, Co, Ni, etc.), and *x*, *y*, and *n* represent the contents of alkaline metal ions, [M′(CN)_6_] vacancies, and crystal water (including ligand water which occupies the [M′(CN)_6_] vacancy sites and is chemically bonded with the unsaturated M ions and zeolite water which is located in the interstitial sites), respectively. The 3D open and flexible framework of PBAs enable the reversible storage of large K-ions. More intriguingly, PBAs seem to be the only cathode candidate which shows a preference for K compared with Li and Na. The better fit of K-ions in the cavities of PBAs stabilizes the structure, leading to higher redox voltage and more stable cycling performance in KIBs over LIBs and NIBs^5,16^.

Among the various types of PBAs reported so far, potassium-rich manganese hexacyanoferrate (K_*x*_Mn[Fe(CN)_6_]_1−*y*_□_*y*_•*n*H_2_O,) features the largest achievable capacity and highest average working voltage along with facile synthesis and abundant elements, making it one of the most distinguished cathode materials for KIBs. Previous work has demonstrated the great potentials of this material towards K-ion storage. For instance, Goodenough et al. reported K_1.89_Mn[Fe(CN)_6_]_0.92_•0.75H_2_O as the cathode material for KIBs, which could deliver a high capacity of 142 mAh g^−1^ with two close voltage plateaus centered at 3.6 V (vs. K^+^/K), corresponding to a specific energy of 511 Wh kg^−1^ ^11^. Almost at the same time, Komaba et al. reported K_1.75_Mn[Fe(CN)_6_]_0.93_•0.16H_2_O cathode for KIBs^12^. It delivered a discharge capacity of 141 mAh g^−1^ with a specific energy of 536 Wh kg^−1^, which exceeds that of LiFePO_4_. Nevertheless, the reported K_*x*_Mn[Fe(CN)_6_]_1−*y*_□_*y*_•*n*H_2_O still contains a considerable amount of [Fe(CN)_6_] vacancies and water, which originate from the ultra-fast nucleation of the sample in aqueous solutions. The [Fe(CN)_6_] vacancies will cause K-deficiency in the cavities for the reason of charge balance and the empty cavities are usually occupied by zeolite water molecules. Meanwhile, [Fe(CN)_6_] vacancies also generate exposed Mn ions, which are susceptible to be coordinated by water molecules, further increasing the water content in the material. The K-deficiency will reduce the specific capacity and the interstitial water usually leads to side reactions, both of which shorten the lifetime of K_*x*_Mn[Fe(CN)_6_]_1−*y*_□_*y*_•*n*H_2_O.

In present work, a simple and practically scalable chelating agent assisted precipitation method is introduced to fabricate K_2_Mn[Fe(CN)_6_]. The obtained sample is nearly stoichiometric with an extremely low content of defects and water. In K-metal cells, it presents exceptional electrochemical performance. Specifically, it could deliver a reversible capacity of 154.7 mAh g^−1^ and an average discharge voltage of 3.941 V (vs. K^+^/K), leading to a high specific energy of 609.7 Wh kg^−1^ with an impressive round-trip efficiency of 96%. This material also exhibits good cycling stability at both low and high specific currents. More encouragingly, over 80% of its capacity can be retained after 7800 cycles, which is the best long-term cycle life among all the reported cathode materials for KIBs. Experimental investigations combined with theoretical calculations reveal that the eliminated defects and negligible water in the sample play critical roles in stabilizing its cycling performance. Finally, the prototype potassium-ion full-cell consisting of the obtained cathode and graphite anode could demonstrate a specific energy as high as 331.5 Wh kg^−1^ (based on the total mass of cathode and anode materials) and negligible capacity loss after 300 cycles, as well as remarkable rate performance (58% capacity retention at 6.67 C vs. 0.1 C), promising the practical application of KIBs as a low-cost and stable energy storage system.

## Results

### Materials synthesis and characterizations

**Fig. 1:**
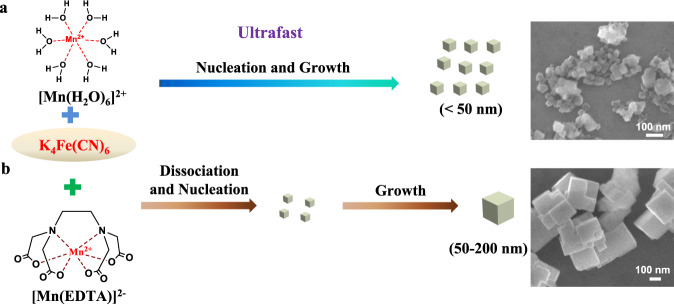
K_2_Mn[Fe(CN)_6_] synthetic pathways. Co-precipitation synthesis with no chelating agent (**a**) and with EDTA chelating agent (**b**).

Conventionally, the synthesis of K_2_Mn[Fe(CN)_6_] is based on the direct co-precipitation reaction among Mn^2+^, [Fe(CN)_6_]^4-^, and K^+^ in an aqueous solution, as schematically illustrated in Fig. 1a^11,12,17^. Because of the extremely small solubility product constant of K_2_Mn[Fe(CN)_6_] (*K*_sp_ = 10^−12.1^)^18^, the co-precipitation reaction occurs expeditiously under an uncontrollable way. As shown by the powder X-ray diffraction (XRD), scanning electron microscopy (SEM), and transmission electron microscopy (TEM) results in Supplementary Figs. 1, 2, the resultant sample (denoted as KMF-C hereafter) is in monoclinic phase (P21/n symmetry, *a* = 10.14201 Å, *b* = 7.25722 Å, *c* = 7.03956 Å, and *β* = 90.52°, Supplementary Table 1) consisting of nonuniform and aggregated nanoparticles (<50 nm). A combination of the inductively coupled plasma mass spectrometry (ICP-MS) (Supplementary Table 2) and thermogravimetric analysis (TGA) (Supplementary Fig. 3) demonstrate that KMF-C contains a high content of [Fe(CN)_6_] vacancies (8 at.% vs. Mn) and water (7.51 wt.%), equivalent to a calculated chemical formula of K_1.72_Mn[Fe(CN)_6_]_0.92_□_0.08_•1.43H_2_O, consistent with the results reported in the literature^11,12^. The TGA-MS (mass spectrometry) (Supplementary Fig. 4) result also indicates that KMF-C will release toxic HCN at 260˚C, suggesting the loss of -CN- linkers and collapse of the framework at high temperature.

To obtain high quality and low defect K_2_Mn[Fe(CN)_6_] sample, ethylenediaminetetraacetic acid dipotassium salt (EDTA-2K) is adopted as the chelating agent to control the crystallization process as illustrated in Fig. 1b. It is noted that compared with the widely used citrate-based chelating agent, which has been recently reported by Pasta et al.^19^, EDTA^4-^ exhibits a much stronger complex ability towards Mn^2+^ (K_stable_[Mn(EDTA)]^2−^ = 10^13.8^ » *K*_stable_[Mn(citrate)]^−^ = 10^3.67^)^18,19^. Hence, the [Mn(EDTA)]^2−^ will act as the reservoir to slowly release Mn^2+^ during the precipitation reaction. Consequently, the nucleation and growth of K_2_Mn[Fe(CN)_6_] can be greatly suppressed, which will result in an sample (denoted as KMF-EDTA) with significantly reduced defects and water. According to the published work^20^, the reactant concentration of 0.04 M and arbitrarily chosen reaction time of 4 h are used in this work which result in a product yield of 74% (see Materials Synthesis section in the Supplementary Information for detailed synthesis procedure). It is later discovered that the reaction time could be further reduced for scale up synthesis because of the fast reaction between the reactants (vide infra).

**Fig. 2:**
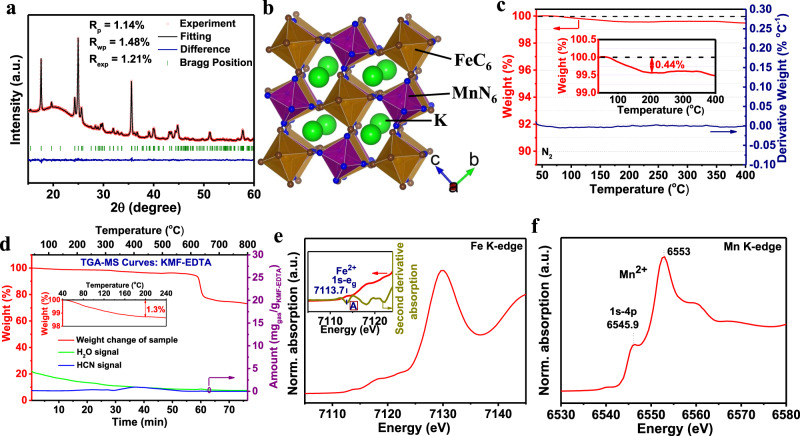
Characterizations of KMF-EDTA. **a**, **b** Rietveld refinement XRD pattern (**a**) and the schematic crystal structure (**b**) of the KMF-EDTA sample. **c** TGA curve of KMF-EDTA sample tested in N_2_. **d** TGA-MS curves of the KFM-EDTA sample tested in N_2_ after the sample is stored in room atmosphere (~25 ˚C, relative humidity:40–60%) for 14 days. Note: the sample is stored immediately after the synthesis. **e**, **f** XANES spectra of the KMF-EDTA sample for the **e** Fe and **f** Mn K-edges. The inset in **e** shows the second derivative for the pre-edge region of the Fe K-edge spectrum. See Supplementary Fig. 7 and the corresponding text for the analysis of the oxidation state of Fe and Mn in the KMF-EDTA sample.

The crystal structure of the as-synthesized KMF-EDTA is examined by powder XRD (Fig. 2a) and Rietveld refinement of the XRD pattern indicates a monoclinic structure (P21/n symmetry, *a* = 10.0912 Å, *b* = 7.3243 Å, *c* = 6.9442 Å, and *β* = 90.02°) with atomic positions listed in Supplementary Table 3, similar with the previous reports^12,21^. The corresponding crystal structure of KMF-EDTA is illustrated in Fig. 2b, revealing a distorted open framework with C-coordinated Fe cations and N-coordinated Mn cations^21^. The ICP-MS analysis (Supplementary Table 2) gives a K:Mn:Fe molar ratio of 1.94:1:0.994, suggesting a high K-ion content and exceedingly low [Fe(CN)_6_] vacancies (0.6 at.% vs. Mn). TGA is applied to estimate the water content in the sample. As shown in Fig. 2c, the KMF-EDTA sample exhibits a negligible weight loss under 100˚C and a considerably low weight loss (~0.44 wt.%) from 100 to 200 ˚C, which can be ascribed to the evaporation of weakly coordinated ligand water^8^, implying that this sample does not require extensive vacuum dehydration before cell assembling. Fourier-transform infrared (FT-IR) spectra of the KMF-EDTA sample (Supplementary Fig. 5) shows a single peak around 2067 cm^−1^, which is ascribed to the vibration of Mn^2+^-N ≡ C-Fe^2+^ bonds^22^. The characteristic signals of water molecules are absent in the FT-IR spectra, further bearing out the extremely low residual water content in the KMF-EDTA. As shown by the TGA-MS result in Fig. 2d, even after stored in room atmosphere for 14 days, KMF-EDTA only demonstrates a slightly increased water uptake (~1.3 wt.%) compared with the as-synthesized sample. Furthermore, negligible release of HCN is detected for KMF-EDTA during heating, indicating that this material preserves its intact structure. The thermal stability of the KMF-EDTA is further characterized by recording its XRD patterns as a function of temperature. As shown in Supplementary Fig. 6, upon heating in N_2_ atmosphere to 300 ˚C, the XRD diffraction signals of KMF-EDTA do not display detectable variations, suggesting its robust crystal structure.

On the basis of the ICP-MS and TGA results, the chemical formula of KMF-EDTA is calculated as K_1.94_Mn[Fe(CN)_6_]_0.994_□_0.006_•0.08H_2_O (Supplementary Table 2), very close to the theoretical stoichiometry of K_2_Mn[Fe(CN)_6_] and possessing a lower content of defects and water than that of the KMF sample (denoted as KMF-Citrate) which was synthesized using citrate as the chelating agent (Supplementary Table 4)^19^. The X-ray absorption near edge structure (XANES) spectra of Fe and Mn K-edges for the KMF-EDTA (Fig. 2e, f) verify that both Fe and Mn are at the oxidation state of +2 (see Supplementary Fig. 7 and the corresponding text)^23,24^. The SEM and TEM images reveal that the KMF-EDTA sample possesses a cubic shape with edge length of 50–200 nm (Supplementary Fig. 8). As compared in Supplementary Fig. 9 and Supplementary Table 4, KMF-EDTA outperforms almost all of the reported K_*x*_Mn[Fe(CN)_6_]_1−*y*_□_*y*_•*n*H_2_O samples, in terms of the K-ion content, [Fe(CN)_6_] vacancies, and water content.

UV–vis spectroscopy is used to investigate the effect of EDTA on the precipitation kinetics of K_2_Mn[Fe(CN)_6_] (Supplementary Fig. 10 and the corresponding text). The intensity of the characteristic absorption signal of K_4_Fe(CN)_6_ in the aqueous solution at 327 nm wavelength is monitored during the precipitation. As shown in Supplementary Fig. 10c, the in-situ UV–vis spectrum shows that once adding EDTA-free Mn^2+^ solution into the K_4_Fe(CN)_6_ solution, the absorption intensity of K_4_Fe(CN)_6_ exhibits an abrupt decrease and quickly disappears in 0.5 s. In comparison, it shows a comparatively slow fade rate upon adding the EDTA-containing Mn^2+^ solution and finally vanishes after 2.5 s. Consequently, such a 5-fold extension of the precipitation time can greatly suppress the [Fe(CN)_6_] vacancies from 8% in KMF-C to only 0.6% in KMF-EDTA, enabling more K-ions (1.94 in KMF-EDTA vs. 1.72 in KMF-C) in the lattice^25^, which is beneficial for assembling the KIB full-cells with a potassium-free anode (e.g., graphite). The significantly decreased [Fe(CN)_6_] vacancies and remarkably increased K-ions content also greatly reduce the occupancy sites for ligand water and zeolite water, greatly reducing the water content (0.44 wt.% in KMF-EDTA vs. 7.51 wt.% in KMF-C)^26^, which can minimize the water-related side reactions and enhance the electrochemical performance (vide infra)^10^.

### Electrochemical performance in potassium metal cells

**Fig. 3:**
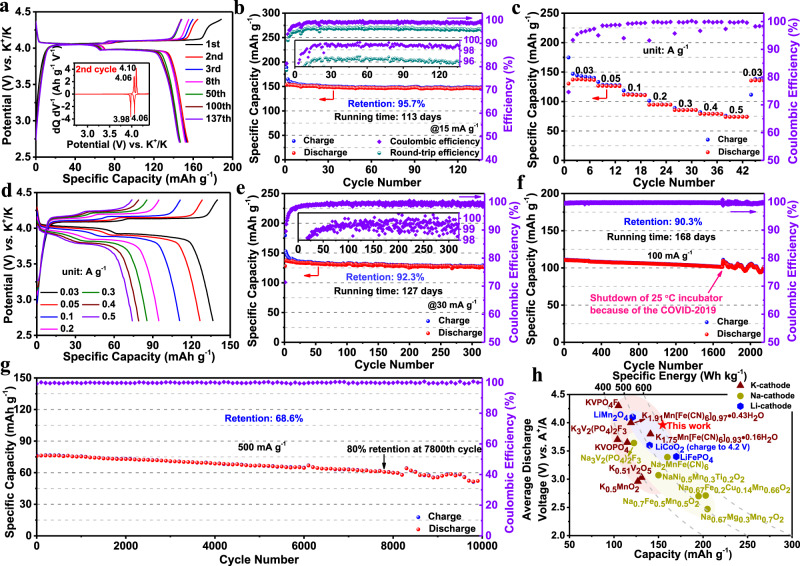
Electrochemical performance of the KMF-EDTA | K metal cells within 2.7–4.4 V (vs. K^+^/K). **a**, **b** Galvanostatic charge–discharge voltage profiles (**a**) and cycling performance at 15 mA g^−1^ (~0.1 C) (**b**). The inset in **a** shows the differential capacity (dQ dV^−1^) curve of the 2^nd^ cycle. The inset in **b** shows the enlarged view of efficiency, including the coulombic efficiency and round-trip efficiency. **c**, **d** Rate capability (**c**) and voltage profiles (**d**) cycled at different specific currents from 30 mA g^−1^ to 500 mA g^−1^. **e**–**g** Long-term cycling performance at **e** 30 mA g^−1^, **f** 100 mA g^−1^, and **g** 500 mA g^−1^, respectively. The inset in **e** shows the enlarged view of Coulombic efficiency. **h** Average discharge voltage and specific energy versus specific capacity of some reported cathodes for LIBs, NIBs, and KIBs. The capacity and specific energy are calculated based on the mass of cathode materials in the corresponding metallic Li/Na/K cells. The data used for plotting (**h**) are listed in Supplementary Table 5.

The electrochemical performances of the samples are examined in potassium metal cells within 2.7–4.4 V (vs. K^+^/K) with our previously reported non-flammable phosphate-based electrolyte (2.5 M potassium bis(fluorosulfonyl) imide (KFSI) salt dissolved in triethyl phosphate (TEP) solvent), which exhibits an excellent anodic stability (Supplementary Fig. 11 and the corresponding text) and is also well-compatible with the graphite anode^27,28^. For the following laboratory evaluation of the electrochemical performance of the samples in the coin-type cells, a typical active material loading of 1 mg cm^−2^ is used. Figure 3a shows the galvanostatic charge–discharge potential profiles of KMF-EDTA at 15 mA g^−1^ (0.1 C, 1 C = 150 mA g^−1^). During the first charge (depotassiation) process, two distinct voltage plateaus centered at 4.06 and 4.10 V (vs. K^+^/K) are observed. The subsequent 1^st^ discharge (potassiation) process displays two corresponding reduction potential plateaus centered at 3.98 and 4.06 V (vs. K^+^/K). These two pairs of well-defined redox plateaus at 4.06/3.98 and 4.10/4.06 V (vs. K^+^/K), which are attributed to low-spin Fe^2+/3+^ and high-spin Mn^2+/3+^ redox couples, respectively^11,12^, maintain almost unchanged over 100 cycles. Remarkably, the potential hysteresis for the two redox couples is only 80 and 40 mV, which are the smallest among all reported PBAs electrode materials^29^. As shown in Fig. 3a, the first charge and discharge capacities of KMF-EDTA are 188.8 and 153.5 mAh g^−1^ with an initial Coulombic efficiency of 81.34%. The first charge capacity of KMF-EDTA exceeds its theoretical value (~156 mAh g^−1^, corresponding to full depotassiation from K_2_Mn[Fe(CN)_6_]). Considering the negligible water content in the sample, the excess capacity should be ascribed to the oxidative decomposition of the electrolyte at high voltages, as evidenced by the small voltage tail at the end of the 1^st^ charge process. After the 1^st^ cycle, the charge capacity gradually decreases from 164.7 mAh g^−1^ at the 2^nd^ cycle to 155.6 mAh g^−1^ at the 8^th^ cycle and the Coulombic efficiency approaches 98%, indicating that the electrolyte decomposition only occurs during the initial few cycles.

Figure 3b reveals the cycling performance of the KMF-EDTA cathode at 15 mA g^−1^. Upon cycling, the material is capable to deliver a reversible capacity of ~154.7 mAh g^−1^ (at the 2^nd^ cycle). It also demonstrates an outstanding cycling stability with a remarkably high capacity retention of 95.7% after 137 cycles (testing period ~113 days, 2712 h). Such a low capacity decay rate at this harsh low current rate (~0.1 C) testing condition is impressive, which has never been achieved in the literature for the PBAs-based cathode materials in non-aqueous KIBs. As shown in Fig. 3b, the Coulombic efficiency increases from 94% at the 2^nd^ cycle to 99.1% at the 16^th^ cycle, and maintains an average value of 99.2% during the following cycles, verifying a highly reversible potassiation–depotassiation process. The high Coulombic efficiency along with the ultra-low voltage hysteresis enables an extremely high round-trip efficiency (~96%, Fig. 3b), comparable to that of state-of-the-art LIBs^30,31^. For the practical applications, the Coulombic efficiency of the sample should be further enhanced to the ideal value (~100%) by optimizing the compositions of the electrolyte or other approaches.

As shown in Supplementary Fig. 12a, the KMF-EDTA can preserve a considerably stable average discharge potential around 3.941 V (vs. K^+^/K), manifesting its highly stable redox reactions. Owing to its high discharge capacity and high redox voltage, the KMF-EDTA can deliver a specific energy as high as 609.7 Wh kg^−1^ (calculated on the basis of the discharge voltage and specific capacity during the 2^nd^ cycle) (Fig. 3h, Supplementary Fig. 12b, Supplementary Table 5), which is so far the highest value for the reported cathode materials of KIBs. This value even outperforms some commercial cathode materials for LIBs (for example LiFePO_4_, LiCoO_2_ (charge to 4.2 V vs. Li^+^/Li), LiMn_2_O_4_) and stellar cathode materials for NIBs (Na_2_Mn[Fe(CN)_6_], Na_0.67_Fe_0.2_Cu_0.14_Mn_0.66_O_2_, Na_3_V_2_(PO_4_)_2_F_3_, etc.)^4,32–35^. In addition, the discharge capacity above 3.8 V (vs. K^+^/K) comprises ~91% of its total discharge capacity, which is beneficial for the practical implementation of this material in high voltage KIBs.

As a comparison, the electrochemical performance of KMF-C sample with a high content of defects and water is also tested. It merely delivers an initial discharge capacity of 131 mAh g^−1^ at 15 mA g^−1^ (Supplementary Fig. 13a), lower than that of KMF-EDTA (Fig. 3a). It also exhibits inclined voltage plateaus with much larger potential hysteresis (120 and 150 mV), lower Coulombic efficiency (around 97%), and inferior cycling stability with only 81.8% capacity retention after 100 cycles (Supplementary Fig. 13). Undoubtedly, both defects and water in KMF-C are detrimental to its electrochemical performance.

In addition to its high reversible capacity and high redox voltage, the KMF-EDTA also displays good rate capability (Fig. 3c, d). It is capable to deliver reversible capacities of 136.7, 126.6, 111.6, 94.9, 85.5, 79, and 74 mAh g^−1^ at the specific currents of 0.03, 0.05, 0.1, 0.2, 0.3, 0.4, and 0.5 A g^−1^, respectively. When the current is set back to 0.03 A g^−1^, the capacity can recover to the value of 136 mAh g^−1^, manifesting the robust structural stability of the KMF-EDTA. To further assess the rate performance of KMF-EDTA and avoid the K dendrite growth at high currents, the rate capability test is performed under a constant charge specific current of 30 mA g^−1^. Under such charge–discharge conditions, discharge capacity of 81 mAh g^−1^ can be acquired even at 1.0 A g^−1^, corresponding to 59.3% capacity retention when the discharge current experiences a 33-fold increase (from 0.03 A g^−1^ to 1.0 A g^−1^) (Supplementary Fig. 14). Such a remarkable rate capability should be ascribed to the fast migration kinetics of K-ion in the Mn[Fe(CN)_6_] framework^11,12^.

As shown in Fig. 3e and Supplementary Fig. 15, the KMF-EDTA provides a reversible capacity of ~136 mAh g^−1^ and excellent cycling stability with 92.3% capacity retained after 320 cycles (corresponding to a testing period of 127 days, 3048 h) at 30 mA g^−1^ (0.2 C). Furthermore, it also demonstrates superb long-term cycling stability at 100 and 500 mA g^−1^, respectively. At a moderate specific current of 100 mA g^−1^ (0.67 C), the KMF-EDTA can deliver a reversible capacity of 110 mAh g^−1^ with a capacity retention of 90.3% even after 2150 cycles (running time over 168 days, 4032 h), with only 0.0045% capacity decay per cycle (Fig. 3f, Supplementary Fig. 16). Note that the fluctuation in Fig. 3f after 1700 cycles originates from the temperature variation during the test (Supplementary Fig. 17), which was caused by the shutdown of the 25˚C incubator due to the outbreak of Covid-19. At a high specific current of 500 mA g^−1^ (~3.33 C), a reversible capacity of 76.3 mAh g^−1^ is acquired for KMF-EDTA (Fig. 3g, Supplementary Fig. 18) with 80% capacity retention after 7800 cycles and 69% capacity retention after 10000 cycles. The superior cycle life of the KMF-EDTA should be attributed to its highly stable structure, as evidenced by the well-preserved crystal structure and morphology of the cycled electrode (Supplementary Fig. 19).

### K-ion storage mechanism

In order to explore the structure evolution of KMF-EDTA during K-ion insertion and extraction, ex situ XRD test is conducted, as shown in Supplementary Fig. 20. When KMF-EDTA is charged to the midpoint between the two potential plateaus, a new set of peaks (2*θ* = 17.1, 24.1, 34.2˚) corresponding to the cubic structure appear at the expense of the peaks (2*θ* = 17.4, 24.8, 35.4˚) of the pristine KMF-EDTA^11,12^. At the end of the charge, the cubic phase completely transforms to tetragonal structure^12^. During the following K-ion insertion process, a reverse tetragonal–cubic–monoclinic phase transition occurs and all the peaks can recover to their original position at the end of discharge, suggesting that the excellent structural reversibility of KMF-EDTA during the extraction-insertion of K-ions.

**Fig. 4:**
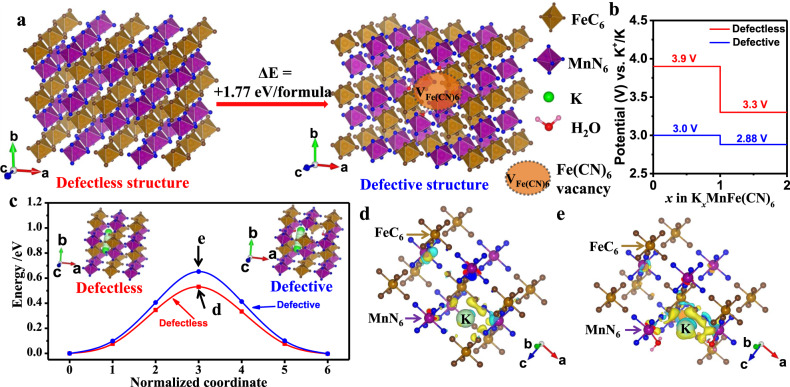
First-principles calculations. **a** The calculated formation energy of the defective structure with defects and water in KMF. The K atoms and C≡N bonds are omitted for clarity. **b** The calculated voltage plateaus for the KMF with defectless and defective structures. **c** Migration energy barriers of the K-ions diffusion within the lattice of the KMF with defectless and defective structures. The insets in **c** show the calculated K-ion migration paths. **d**, **e** The differential charge density diagram of the **d** defectless structure and **e** defective structure in the transition state marked in **c**. The yellow and cerulean region represent the electron-rich and electron-poor area, respectively. The corresponding valence states of K ion in **d** and **e** are listed in Supplementary Table 6.

First-principles density functional theory (DFT) calculation is then used to further elucidate the K-ion storage mechanism in KMF-C and KMF-EDTA. First, the formation energy of KMF-C with defects and water is estimated to be 1.77 eV/formula higher than that of KMF-EDTA (Fig. 4a), suggesting the thermodynamically more unstable structure of KMF-C than that of KMF-EDTA. Second, as shown in Fig. 4b, the calculated voltage for K-ion insertion is higher for KMF-EDTA than that for KMF-C, which is in qualitative agreement with the experimentally obtained equilibrium potentials by the galvanostatic intermittent titration technique (Supplementary Fig. 21). Third, the calculation also indicates a higher K-ion diffusion energy barrier in KMF-C than that in KMF-EDTA (0.65 eV vs. 0.53 eV) (Fig. 4c), which originates from the stronger interaction between K-ion and the defective host structure in KMF-C as evidenced by the differential charge density diagram (Fig. 4d, e, Supplementary Table 6 and the corresponding text). Thus, both the thermodynamic and kinetic origins are expected to account for the observed lower discharge potential for KMF-C. Previous calculation indicates that the binding strength of H_2_O with the lattice will be significantly weakened as Na-ion is extracted from Na_x_MnFe(CN)_6_•nH_2_O, which enable water to escape during the Na-ion extraction process^36^. Such electrochemical dehydration was shown to raise the redox potentials in Na_x_MnFe(CN)_6_•nH_2_O^37^. Similar phenomenon is also expected to take place in KMF-C, as evidenced by the variation of its differential capacity (dQ dV^−1^) curve upon cycling (Supplementary Fig. 13d) and further supported by the upshift of the charge–discharge potential of KMF-C after the sample is dehydrated (Supplementary Fig. 22). The escaped water from KMF-C will induce side reactions, causing its capacity degradation and low Coulombic efficiency. Moreover, DFT calculation also indicates the binding strength towards Mn-ion is stronger for the currently used TEP solvent than that for water (binding energy: 0.52 eV for Mn-TEP vs. 0.09 eV for Mn-H_2_O). Hence, once ligand water molecules escape from the lattice, the remaining exposed Mn-ion at the defect sites is susceptible to be coordinated by the TEP solvent and dissolve into the electrolyte. The ICP-MS results (Supplementary Table 7) bear out that the dissolved Mn content is much higher in the KMF-C cell than that in the KMF-EDTA cell (4 mg L^−1^ for KMF-EDTA vs. 11 mg L^−1^ for KMF-C) after 100 cycles. Self-discharge tests of two samples (Supplementary Fig. 23) show that 92% of its discharge capacity can be achieved for KMF-EDTA after rest for 120 h at the fully charged state while this value is 86% for KMF-C. As shown in Supplementary Fig. 23, the capacity loss of KMF-C is mainly caused by the reduced capacity from the high potential plateau corresponding to the Mn^2+^/Mn^3+^ redox couple, due to the dissolution of Mn-ion into the electrolyte.

Based on the above experimental and theoretical investigations, it can be concluded that the underlying reasons for the inferior electrochemical performance of KMF-C compared with KMF-EDTA are as follows: (1) the Fe(CN)_6_ vacancies and water in KMF-C cause higher K-ion transport barrier, resulting in the increased voltage polarization;^25,38^ (2) the escape and subsequent decomposition of H_2_O occurs at high potentials in KMF-C, leading to side reactions;^10^ (3) the severe dissolution of Mn-ion in KMF-C, causing the poor cycling stability;^39^ (4) other reasons, such as the [Fe(CN)_6_] vacancies induced the unevenly varied Mn-N bond length during Jahn–Teller distortion (see Supplementary Fig. 24 and the corresponding text)^37^, may also contribute to the capacity degradation of KMF-C.

### Electrochemical performance of K-ion full-cells

**Fig. 5:**
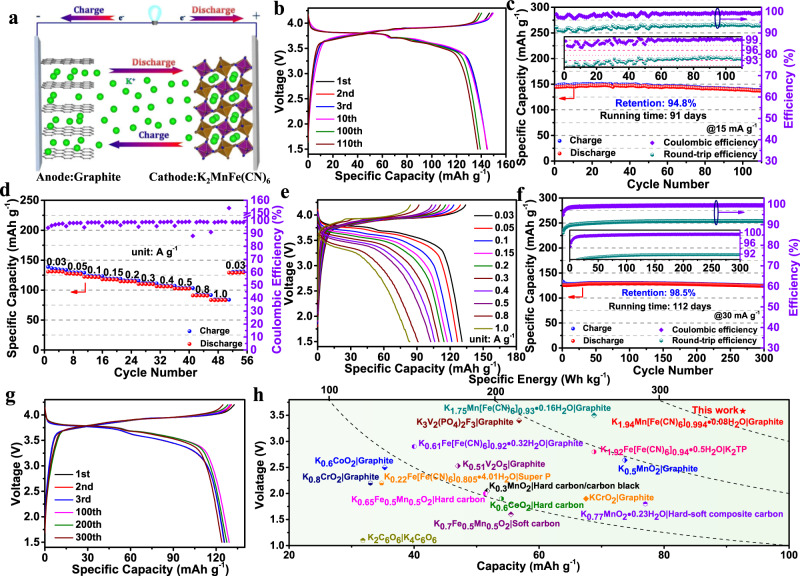
K-ion full-cell performances in the 1.5–4.2 V voltage range. **a** A schematic illustration of the K-ion full cell. **b**, **c** Galvanostatic charge–discharge voltage profiles (**b**) and cycling performance (**c**) of the KMF-EDTA | | Cyc-graphite full-cell at 15 mA g^−1^ (~0.1 C). **d**, **e** Rate capability (**d**) and voltage profiles (**e**) of the KMF-EDTA | | Cyc-graphite full-cell under a constant charge specific current (0.03 A g^−1^) with different discharge specific currents from 0.03 to 1.0 A g^−1^. **f**, **g** Long-term cycling stability (**f**) and voltage profiles (**g**) of the KMF-EDTA | | Cyc-graphite full-cell at 30 mA g^−1^. The insets in **c** and **f** show the enlarged view of efficiency. The specific current and specific capacity in **b**–**g** are calculated based on the mass of KMF-EDTA in the full-cell. **h** Average discharge voltage and specific energy versus capacity of some reported K-ion full-cells. Note: both the capacity and specific energy in **h** are calculated based on the total mass of the cathode and anode materials in the corresponding full-cells, and the data used for plotting **h** are listed in Supplementary Table 8.

To evaluate the potential application of the KMF-EDTA cathode in practical KIBs, a prototype K-ion full-cell (denoted as KMF-EDTA | | Graphite) with pristine KMF-EDTA cathode and pristine graphite anode is configured (Fig. 5a) with a negative:positive capacity ratio of 1:1.05 (corresponding to a mass ratio of graphite:KMF-EDTA = 0.6:1). The KMF-EDTA | | Graphite full-cell exhibits a charge capacity of 179.3 mAh g^−1^ and discharge capacity of 130.8 mAh g^−1^ with an average discharge voltage of 3.27 V during its 1^st^ charge–discharge cycle (Supplementary Fig. 25). However, the discharge capacity drops to 103.8 mAh g^−1^ after 5 cycles. Detailed investigation indicates that although the graphite anode could deliver a reversible capacity of ~261.7 mAh g^−1^ with good cycling stability and rate capability, its Coulombic efficiency during the initial several cycles of SEI formation is insufficient (Supplementary Fig. 26), leading to the capacity decay in Supplementary Fig. 25. Therefore, the graphite anode is pre-cycled for 12 times to eliminate the effect of SEI and then assembled with pristine KMF-EDTA cathode into the full-cell (denoted as KMF-EDTA | | Cyc-graphite). As shown in Fig. 5b, the obtained KMF-EDTA | | Cyc-graphite full-cell can stably operate between 1.5 and 4.2 V at a low specific current of 15 mA g^−1^ (0.1 C, based on the mass of the KMF-EDTA in the full-cell). After the initial few activation cycles, it is capable to deliver a high capacity of 145 mAh g^−1^ at 15 mA g^−1^ with an average discharge voltage of 3.58 V (Supplementary Fig. 27). The average discharge voltage of the KMF-EDTA | | Cyc-graphite full-cell (3.58 V) is even higher than that of the commercial LiFePO_4_ | |Graphite cell (3.2 V)^40^. As shown in Fig. 5c, in spite of testing at a low specific current of 15 mA g^−1^ (~0.1 C), the KMF-EDTA | | Cyc-graphite full-cell presents a decent cycling stability with a high capacity retention of 91% after 110 cycles (running time is over 91 days, 2184 h) and high Coulombic efficiency (~99.3%). During cycling, the Coulombic efficiency and round-trip efficiency is stabilized at the values of 99.1% and 92.8%, respectively, indicating the highly reversible redox reactions at both the graphite anode and KMF-EDTA cathode. It is worth noting that the round-trip efficiency of this cell is much higher than that (75–85%) of the commercially available high-temperature Na-S battery, which is utilized in stationary energy storage^1^. Owing to its high reversible capacity and high average discharge voltage, the prototype KMF-EDTA | | Cyc-graphite full-cell can deliver a specific energy as high as 331.5 Wh kg^−1^ (based on the total mass of the cathode and anode materials), which outperforms all reported K-based full-cells (Fig. 5h, Supplementary Table 8). This value is higher than the specific energy of lead-acid, vanadium redox and nickel metal-hydride batteries and even comparable with the LiFePO_4_ | |Graphite cell (Supplementary Table 9)^40–44^.

Moreover, the KMF-EDTA | | Cyc-graphite full-cell also demonstrates good rate capability. As shown in Supplementary Fig. 28, reversible capacities of 131.5, 115.9, 102.2, 92.9, 85.7, 74.3, and 66.5 mAh g^−1^ can be obtained at specific currents of 0.03, 0.05, 0.1, 0.15, 0.2, 0.3, and 0.4 A g^−1^. It has been reported that the graphite anode possesses excellent high-rate depotasstion capability but poor potassiation capability^6,45^. The poor potassiation capability of the graphite anode limits the charge performance of the full-cell. Therefore, to further examine the discharge rate capability of the full-cell and avoid the potassium dendrite growth during the charge process, the full-cell was tested with a constant charge specific current of 30 mA g^−1^ (~0.2 C) but various discharge rates. As shown in Fig. 5d, e, under such a test condition, the discharge capacities change only from 131.1 to 84.1 mAh g^−1^ when the discharge rate increases from 0.03 to 1.0 (~6.67 C) A g^−1^, bearing out the remarkable high rate discharge capability of the full-cell. Moreover, the full-cell also presents superb cycling stability with 98.5% capacity retention after 300 cycles (running time over 112 days, 2880 h) at 30 mA g^−1^ (~0.2 C, Fig. 5f). Figure 5g presents the corresponding voltage profiles, suggesting the higly reversible charge–discharge process in the full-cell. At a higher specific current (50 mA g^−1^), the full-cell can only demonstrate a capacity retention of 85.6% over 235 cycles (Supplementary Fig. 29a). Compared to the 98.5% capacity retention after 300 cycles at 30 mA g^−1^, this relatively lower capacity retention at the higher specific current of 50 mA g^−1^ is found to be caused by the increased polarization of the graphite anode (Supplementary Fig. 29b–g), which originated from the sluggish potassiation process into graphite^6,45^, emphasizing the necessity of further improvement of graphite anode for practical KIBs. Moreover, the self-discharge behavior of the full-cell is also evaluated. After charged to 4.2 V, the full-cell is left to rest for 120 h. During the rest period, the full-cell’s voltage experiences a sudden drop to 3.9 V initially and then maintains around 3.85 V after 120 h (Supplementary Fig. 30). The subsequent discharge test shows that the full-cell can maintain 94% of its initial capacity, which can be further enhanced by future optimization of the full-cell configuration.

One of the significant safety risk for LIBs is that it must be transported at certain state of charge (~30%) to minimize the possibility of the dissolution of the copper current collector at the anode side^4^. While for KIBs, the aluminum current collector can be used at both the anode and cathode sides. Consequently, the potassium-ion full-cell also possesses one of the crucial safety advantages, i.e., the capability to store and transport at 0 V, indicating a zero energy storage of the cell^4^. To verify its zero voltage storage ability, after being discharged to 1.5 V, the prototype potassium-ion full-cell is held at 0 V for 12 h by a physical short-circuit (Supplementary Fig. 31). Although it is physically short-circuited, the full-cell could fully recover to its full capacity after charge, demonstrating its superb zero voltage storage ability. Overall, the high specific energy of present low-defect and nearly anhydrous K_2_Mn[Fe(CN)_6_] along with its good electrochemical stability and low-cost promises it as an attractive cathode material for practical KIBs and the prototype K-ion full-cell demonstrates great potential as a complementary energy storage system to LIBs for the large-scale energy storage.

## Discussion

In this study, we have synthesized a potassium manganese hexacyanoferrate with an exceedingly low content of defects and residual water with the aid of EDTA and identified it as a promising cathode material for KIBs. The as-prepared KMF-EDTA sample exhibits a high reversible capacity of 154.7 mAh g^−1^, high average discharge voltage (3.941 V vs. K^+^/K), and high specific energy of 609.7 Wh kg^−1^. It also demonstrates superb cycling stability at various specific currents and a capacity retention of 80% is realized after 7800 cycles. Experimental investigations along with theoretical calculations show that the remarkable cycling stability of the obtained sample should be attributed to its defect-free structure with negligible water which effectively mitigates the issues of Mn-ion dissolution and the water-related side reactions. More encouragingly, the prototype KMF-EDTA | | graphite full-cell could deliver a high specific energy of 331.5 Wh kg^−1^ with high round-trip efficiency of 92.8% and negligible capacity decay for 300 cycles, suggesting the great promise of KIBs as the low-cost and high-performance complementary alternative to LIBs for grid-scale energy storage.

## Supplementary information

Supplementary Information

## Data Availability

The data that support the findings of this study are available from the corresponding authors on reasonable request.
