# Supplementary material for: Defect-free potassium manganese hexacyanoferrate cathode material for high-performance potassium-ion batteries
Source: Nat Commun. 2021 Apr 12;12:2167. doi: 10.1038/s41467-021-22499-0 (PMC8041879; doi:10.1038/s41467-021-22499-0)
Supplement: Supplementary file 1 — Supplementary Information [file 41467_2021_22499_MOESM1_ESM.pdf]

Supplementary Information for

Defect-Free Potassium Manganese Hexacyanoferrate Cathode Material for High-Performance Potassium-Ion Batteries

Leqing Deng[1,2,#], Jiale Qu[3,#], Xiaogang Niu[1], Juzhe Liu[1], Juan Zhang[1], Youran Hong[4], Meiying Feng[1], Jiangwei Wang[4], Miao Hu[5], Liang Zeng[6], Qianfan Zhang[3]*, Lin Guo[1,7]*, Yujie Zhu[1,7]*

[1]School of Chemistry, Beihang University, Beijing 100191, P. R. China

[2]School of Physics, Beihang University, Beijing 100191, P. R. China

[3]School of Materials Science and Engineering, Beihang University, Beijing 100191, P. R. China

[4]Center of Electron Microscopy and State Key Laboratory of Silicon Materials, School of Materials Science and Engineering, Zhejiang University, Hangzhou 310027, P. R. China

[5]CNOOC Research Institute of Refining and Petrochemicals, Beijing 102200, P. R. China

[6]Key Laboratory for Green Chemical Technology of Ministry of Education, School of Chemical Engineering and Technology, Tianjin University, Tianjin 300072, P. R. China

[7]Beijing Advanced Innovation Center for Biomedical Engineering, Beihang University, Beijing 100191, P. R. China

[#]These authors contributed equally: Leqing Deng, Jiale Qu

[*]Corresponding authors: yujiezhu@buaa.edu.cn; guolin@buaa.edu.cn; qianfan@buaa.edu.cn

Materials Synthesis

The $K_2Mn[Fe(CN)_6]$ (KMF) samples were obtained by a simple precipitation method and all the reagents used in this work are commercially available. Typically, the KMF-EDTA sample was synthesized under room temperature as follows: 50 mL

of 0.04 M manganese acetate tetrahydrate ($Mn(CH_3COO)_2$•$4H_2O$, Aladdin) solution containing and 0.04 M ethylenediaminetetraacetic acid dipotassium salt dehydrate (EDTA-2K•$4H_2O$, Alfa) was dropwise added into 50 mL of 0.04 M potassium hexacyanoferrate(II) trihydrate ($K_4Fe(CN)_6$•$3H_2O$, Aladdin) solution in ten minutes with continuous magnetic stirring and N_2 bubbling. After aging for 4 h without stirring, the resulting precipitates (yield: 74%) were collected by centrifugation, washed several times with deionized water, and dried under vacuum at 80 ˚C for 10 h. As comparison, the control KMF-C sample was obtained similarly without the addition of EDTA-2K•$4H_2O$.

Materials Characterizations

The powder X-ray diffraction (XRD) data were collected by Rigaku Dmax 2200 X-ray diffractometer with Cu Kα radiation (λ = 1.5416 Å). The morphologies of the samples were recorded by scanning electron microscope (JEOL, JSM-7500F, 5 kV). The TEM images were recorded by Tecnai G2 (FEI, 200 KV). Inductively coupled plasma mass spectrometry (ICP-MS) tests were conducted on an Agilent ICPMS7800. TGA characterization was conducted using a Pyris Diamond thermogravimetric/differential thermal analysis (Perkin Elemer Inc., USA) under N_2 atmosphere with a heating rate of 10 ˚C min^{-1}. TGA-MS was carried out with Pyris 1-Clarus (Perkin Elmer Inc., USA) under N_2 atmosphere with a heating rate of 10 ˚C min^{-1}. FT-IR spectra were carried out with a Nicolet iN10 FT-IR Microscope. XANES measurements were conducted at beamline 1W1B of Beijing Synchrotron Radiation Facility (BSRF). XANES spectrum of the samples was recorded on

transmission model. UV-vis spectra were recorded by a UV-2700 (Shimadzu, Japan). The retrieved electrodes for the XRD and SEM characterizations were obtained by disassembling the cells in the glove box, where they were washed with dry diethyl carbonate to remove the residue electrolyte salts.

Electrochemical Characterizations

The KMF cathode was fabricated by spreading the slurry containing KMF, acetylene black, and polyvinylidene fluoride (PVDF) (weight ratio: 7:2:1) onto the Al foil. The graphite anode was fabricated by spreading the slurry of graphite, acetylene black, and carboxymethyl cellulose sodium (weight ratio: 8:1:1) onto the Al foil. The working electrodes were dried under vacuum at 80 °C overnight. The loading mass of the KMF and graphite on the electrode was ~1 mg cm^{-2}.

The electrolyte used was 2.5 M potassium bis(fluorosulfonyl) imide (KFSI, purity: 99.5%, Ark) dissolved in triethyl phosphate (TEP, TCI), as reported by our group previously.[1] Coin cells (2032-type, MTI Corp.) were assembled in an Ar-filled glovebox (both oxygen and moisture concentrations kept below 0.01 ppm). For the potassium metal cell tests, coin cells were assembled using potassium metal as the counter electrode and a glass microfiber filter (Whatman, Grade GF/D) was used as the separator. Galvanostatic charge-discharge tests were recorded at 25 ˚C with a Land battery testing system (Wuhan LAND electronics, China). The transient voltage versus specifc capacity of the cell is obtained by GITT test at the specific current of 18 mA g^{-1}, consisting of a series of current pulses for 0.5 h, followed by a 4 h relaxation process. To eliminate the irreversible capacity of the graphite anode, the

graphite was initially discharged-charged for 12 cycles at 25 mA g^{-1} between 0.005-2.0 V (vs. K^+/K) in the graphite|K metal cell and then charge to 2.0 V (vs. K^+/K) to obtain the pre-cycled graphite (Cyc-graphite) anode for assembling the KMF-EDTA||Cyc-graphite full-cell. To construct the full-cell, the mass ratio of KMF-EDTA:graphite is set to be 1:0.6. Unless otherwise stated, the calculation of the specific capacities and energy densities for both metallic potassium cells and full-cells was based on the mass of KMF cathode in the cells. The specific energy of the cells is calculated by integration of voltage with the specific capacity which is automatically done by the Land battery testing system. The average discharge voltage was obtained by dividing the specific energy by the specific capacity.

Calculation Methods

First-principles calculations were implemented based on density functional theory (DFT) as implemented in the Vienna ab initio simulation package (VASP) code.[2,3] Projector augmented wave (PAW) pseudopotential was applied and the GGA exchange correlation function was described by Perdew-Burke-Ernzerhof (PBE).[4,5] Kinetic energy cutoff for plane wave expansion was set at 500 eV and the criterion for force convergence during the optimization was -0.02 eV/Å or smaller. Transition-state structures and associated minimum energy pathways for K ion diffusion were computed based on the Climbing Image Nudged Elastic Bond (CI-NEB) method.[6] The formation energy of KMF-EDTA and KMF-C are calculated by[7]:

$$\frac{E_{f-KMF-EDTA}}{formula} = E(K_2MnFe(CN)_6) - 2E(K) - E(Mn) - E(Fe) - 6E(C) - 6E(N)$$

$$\frac{E_{f-KMF-C}}{formula} = E(K_{1.8}Mn[Fe(CN)_6]_{0.94} \cdot 1.5H_2O) - 1.8E(K) - E(Mn) - 0.94E(Fe) - 5.6E(C) - 5.6E(N) - 1.5E(H_2O)$$

Where, $E(K_2MnFe(CN)_6)$ is the energy as calculated. E(K), E(Mn), E(Fe), E(C) and E(N) are the energy of the chemical potential of the single atom, which is energy per atom in a stable pure elemental substance.

Otherwise, the electrochemical voltage profiles for $K_2MnFe(CN)_6$ system were calculated by the following equation:[8]

$$V = -[\frac{E(K_{X2}MnFe(CN)_6) - E(K_{X1}MnFe(CN)_6)}{\Delta x} - E(K)]$$

where V is the average redox potential of $K_XMnFe(CN)_6$ ($0 \leqslant x \leqslant 2$). $E(K_XMnFe(CN)_6$ and E(K) represents the total energy as calculated.

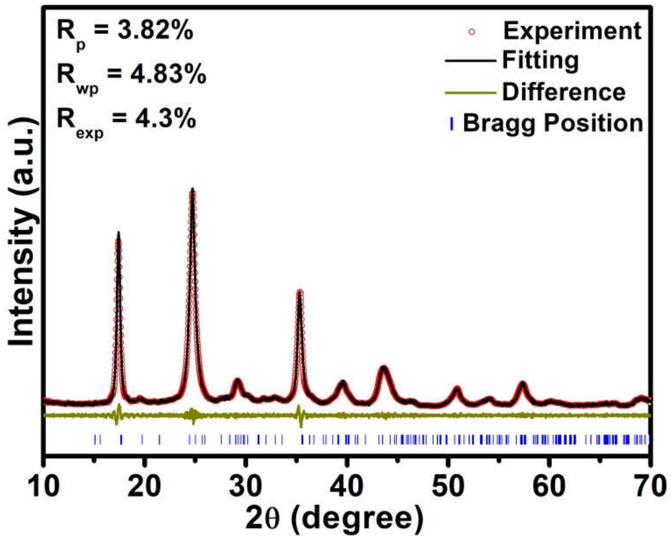

Supplementary Fig. 1 | Rietveld refinement XRD pattern of the KMF-C sample.

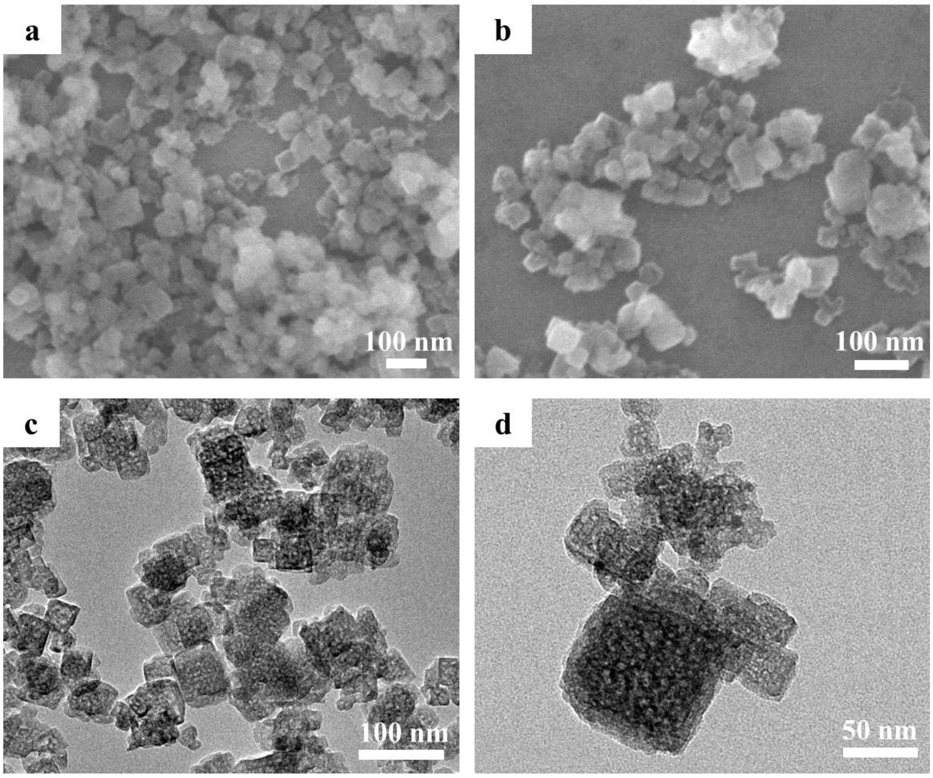

Supplementary Fig. 2 | SEM (**a, b**) and TEM (**c, d**) images of the KMF-C sample.

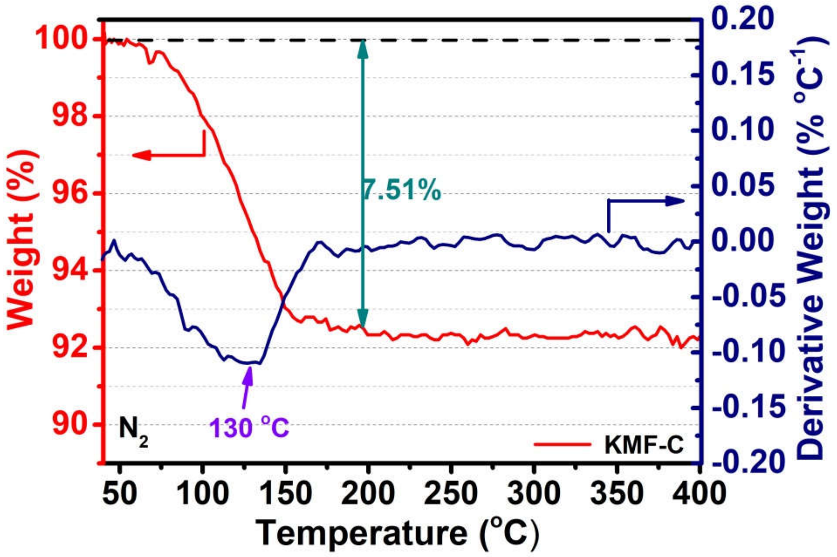

Supplementary Fig. 3 | TGA curve of the KMF-C sample tested in N_2.

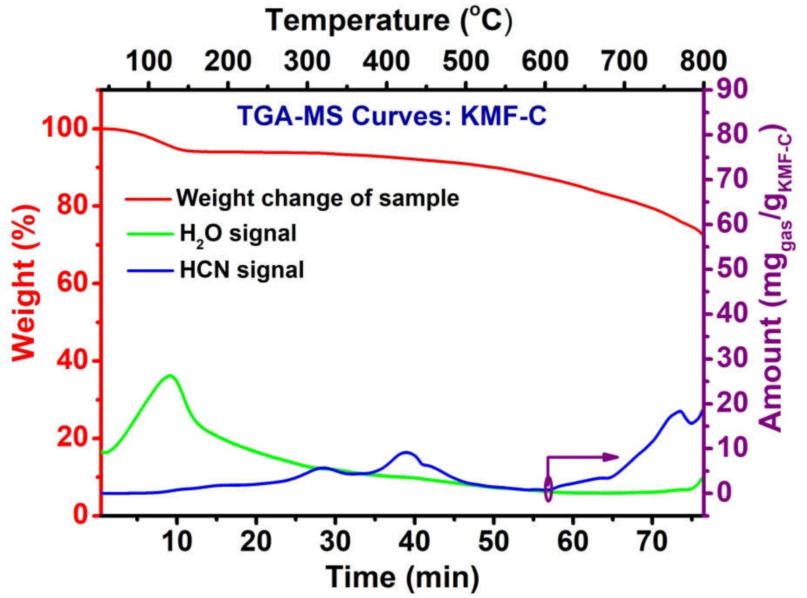

Supplementary Fig. 4 | TGA-MS curves of the KFM-C sample tested in N_2.

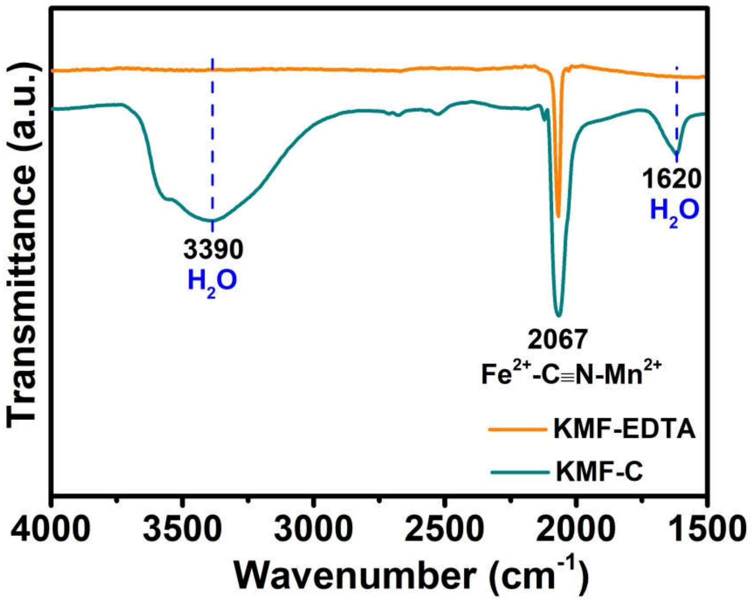

Supplementary Fig. 5 | FT-IR curves of KMF-EDTA and KMF-C.

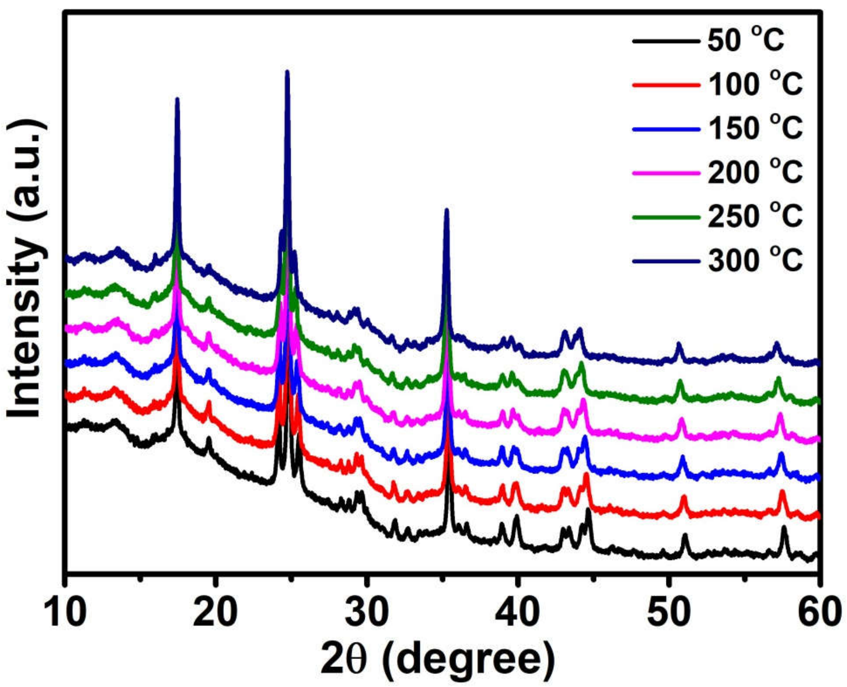

Supplementary Fig. 6 | *In situ* XRD patterns of KMF-EDTA upon heating from 50 to 300 ˚C in N_2.

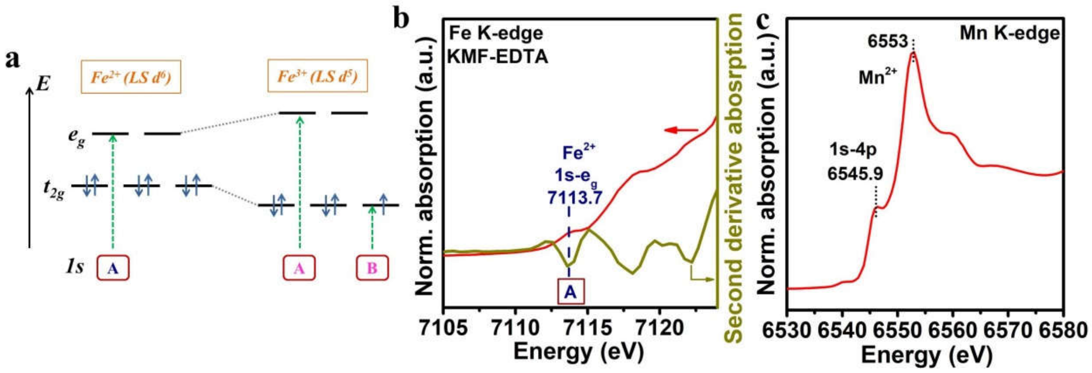

Supplementary Fig. 7 | a Schematic diagram of low-spin (LS) Fe 3d orbitals in the KMF-EDTA sample. **b** Pre-edge region of Fe K-edge spectrum for the KMF-EDTA sample. The parent XANES spectrum from which the pre-edge spectrum is extracted is shown in Fig. 2e. **c** Mn K-edge spectrum for the KMF-EDTA sample with the characteristic $1s$-$4p$ transition and the edge resonance marked by the dotted lines.

The oxidation state of Fe and Mn can be obtained by analyzing the pre-edge region in the Fe and Mn K-edge XANES spectra.[9] Based on the ligand field theory, in spite of the oxidation states, the Fe and Mn atoms in $K_xMn[Fe(CN)_6]$ (x = 0~2) are always at the state of low-spin (LS) and high-spin (HS), respectively.[10,11]

As schematically shown in Supplementary Fig. 7a, for LS Fe^{2+} with a $t_{2g}^6 e_g^0$ electron configuration and spin multiplicity S=0, the only possible $1s$-$3d$ transition in the pre-edge region is the allowed electric quadrupole $1s$-$3de_g$ transition (labeled as process A in the left scheme in Supplementary Fig. 7a) while for LS Fe^{3+} with a $t_{2g}^5 e_g^0$ electron configuration and spin multiplicity S=2 , two transitions are expected, *i.e.*, $1s$-$3dt_{2g}$ (labeled as process B in the right scheme in Supplementary Fig. 7a) and $1s$-$3de_g$ (labeled as process A in the right scheme in Supplementary Fig. 7a).[9,11,12] As shown by the dark yellow line in Supplementary Fig. 7b which is the second derivative of the corresponding absorption curve and depicts the absorption peaks more clearly than the raw absorption curve, only one peak (marked by A) at 7113.7 eV is observed, consistent with the reported values for LS Fe^{2+} of the same family.[11,12] Thus, the XANES spectrum of Fe K-edge unambiguously demonstrates that Fe in KMF-EDTA is at the oxidation state of +2.

Regarding the oxidation state of Mn, as shown in Supplementary Fig. 7c, a single sharp peak at 6545.9 eV is observed in the pre-edge region of the Mn K-edge XANES spectrum for KMF-EDTA. This peak is the fingerprint of Mn^{2+} and attributed to the $1s$-$4p$ transition in Mn^{2+} species of the same family as KMF.[11,13] Besides, according to the reported Mn K-edge spectra of the same family as KMF[11,13], an approximate shift of 5 eV towards higher energy is usually observed due to the oxidation of HS Mn^{2+} to HS Mn^{3+}. Specifically, for HS Mn^{2+}, the reported edge resonance (marked by the dotted line in Supplementary Fig.7c) is around 6553 eV while it is 6558 eV for HS Mn^{3+},[11,13] which can be also used to identify the oxidation state of Mn in KMF. As shown in Supplementary Fig.7c, the edge resonance of the Mn K-edge spectra for KMF-EDTA is around 6553 eV, further demonstrating the Mn^{2+} oxidation state of +2 in the sample.

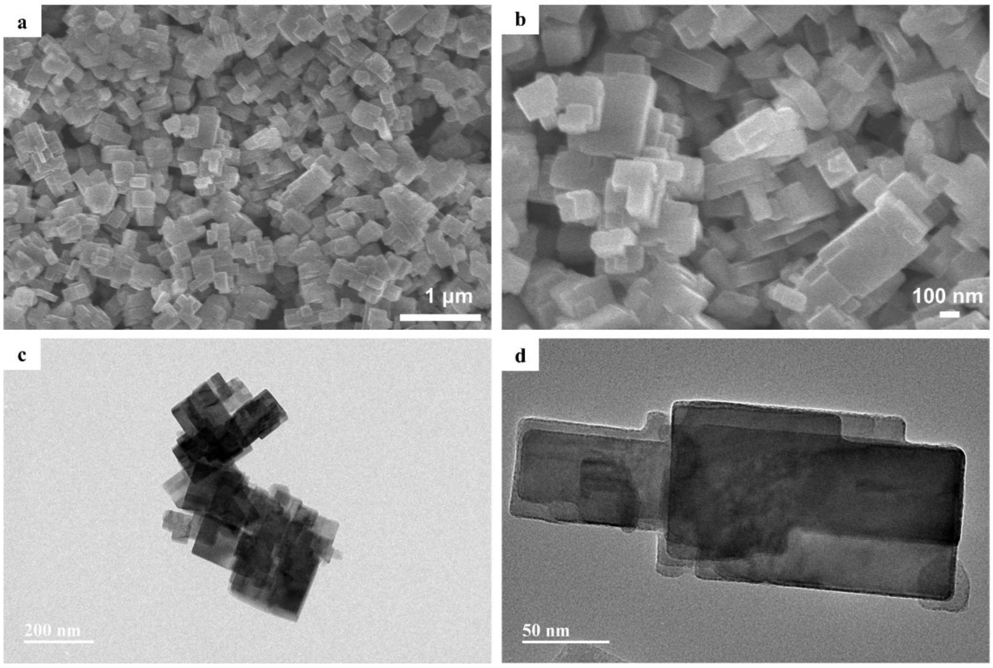

Supplementary Fig. 8 | SEM (**a, b**) and TEM (**c, d**) images of the KMF-EDTA sample.

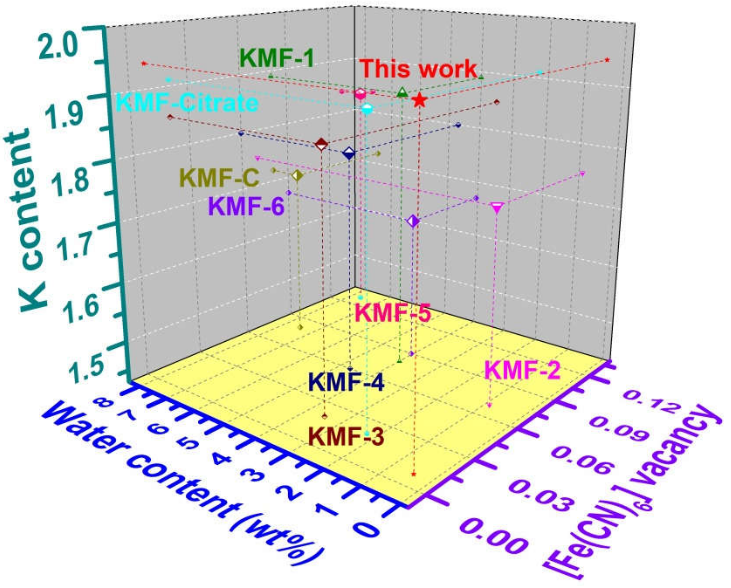

Supplementary Fig. 9 | Comparison of K content, water content, and $[Fe(CN)_6]$ vacancy of some reported KMF. The data used for plotting are listed in Supplementary Table 4.

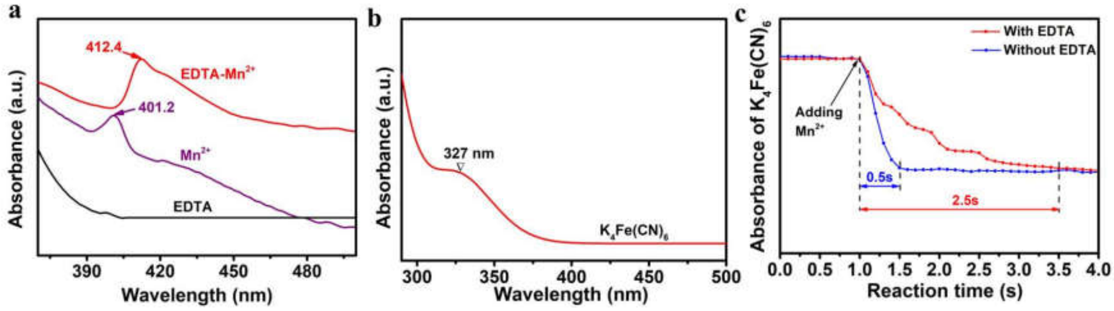

Supplementary Fig. 10 | UV-vis absorption spectra of (**a**) EDTA-2K, Mn^{2+}, EDTA-Mn^{2+} and (**b**) $K_4Fe(CN)_6$. **c** *In situ* UV-vis spectra monitoring the absorption intensity of $K_4Fe(CN)_6$ at 327 nm upon adding Mn^{2+} aqueous solution with or without the EDTA.

As shown in Supplementary Fig. 10a, the blank Mn^{2+} solution shows a maximal absorption at 401.2 nm, while pure EDTA-2K solution exhibits no absorption signal in this area. After adding the EDTA ions into Mn^{2+} solution, the maximal absorption peak shifts to 412.4 nm, suggesting the coordination effect of EDTA with Mn ions. Supplementary Fig. 10b shows the absorption signal of $K_4Fe(CN)_6$ solution with a broad absorption peak centered around 327 nm. The intensity of this absorption signal located at 327 nm wavelength is then recorded for the *in situ* UV-vis spectra to monitor the concentration change of $K_4Fe(CN)_6$ upon adding Mn^{2+} solution with or without the EDTA and the results are shown in Supplementary Fig. 10c.

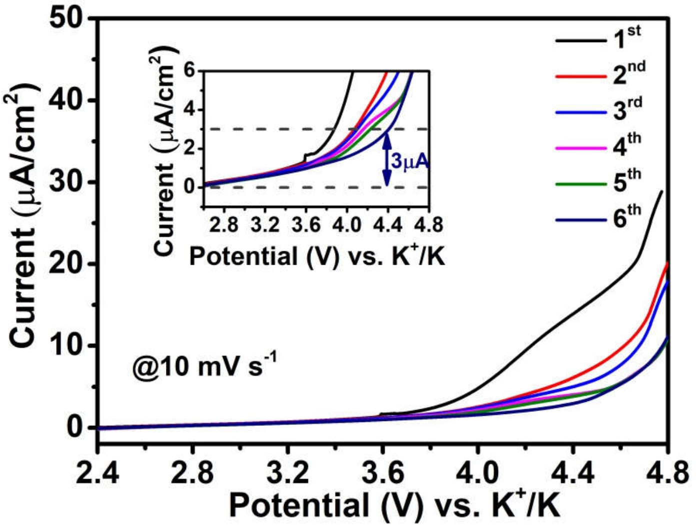

Supplementary Fig. 11 | Linear scanning voltammetry (LSV) curves of the initial six scans at 10 mV s^{-1} in the TEP electrolyte with Al foil as the working electrode and K metal as the counter and reference electrode. The inset shows the enlarged LSV curves from 2.6 V to 4.8 V (*vs.* K^+/K).

Before the electrochemical evaluation of KMF, we examined the anodic stability of the TEP electrolyte used in this work by linear scanning voltammetry (LSV) tests with the K|Al cell and the results are shown in Supplementary Fig. 11. During the 1st scan, the oxidative decomposition of the electrolyte happens starting from 3.6 V (vs. K^+/K) and becomes more and more serious with the increase of the potential (the oxidative current is 13.92 μA/cm^2 at 4.4 V), which results in the small voltage tail at the end of the 1st charge process shown in Fig. 3a in the manuscript. Starting from the 2nd scan, the oxidative current decreases distinctly (6.12 μA/cm^2 at 4.4 V for 2nd cycle, 3μA/cm^2 at 4.4 V for 6th cycle), consistent with the improved Coulombic efficiency (from 81.34% at the 1st cycle, 94% at the 2nd cycle to 99.1% at the 16th cycle) shown in Fig. 3b. It can be seen that the oxidative current experiences a rapid raise when the cell voltage is higher than 4.4 V (*vs.* K^+/K). Thus, to avoid the excessive electrolyte decomposition, the 4.4 V (*vs.* K^+/K) upper cut-off voltage is selected to estimate the K-ion storage performance of KMF.

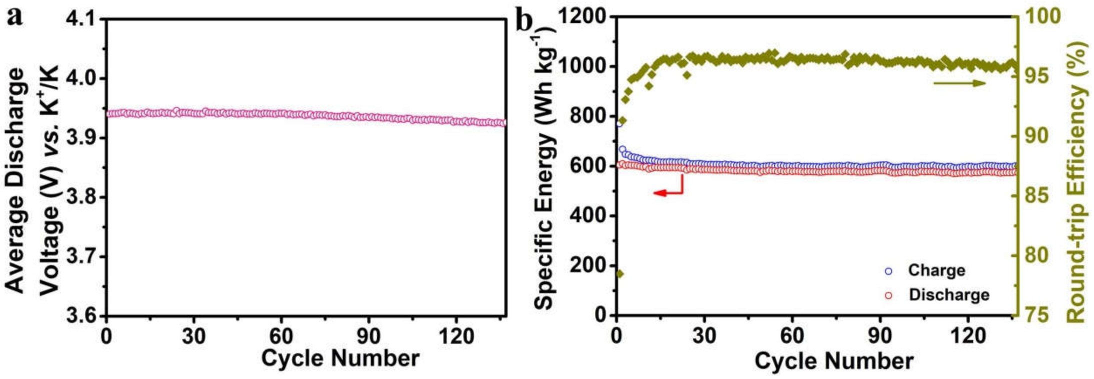

Supplementary Fig. 12 | Average discharge voltage (**a**) and specific energy and round-trip efficiency (**b**) of the KMF-EDTA|K metal cell at 15 mA g^{-1} (0.1C).

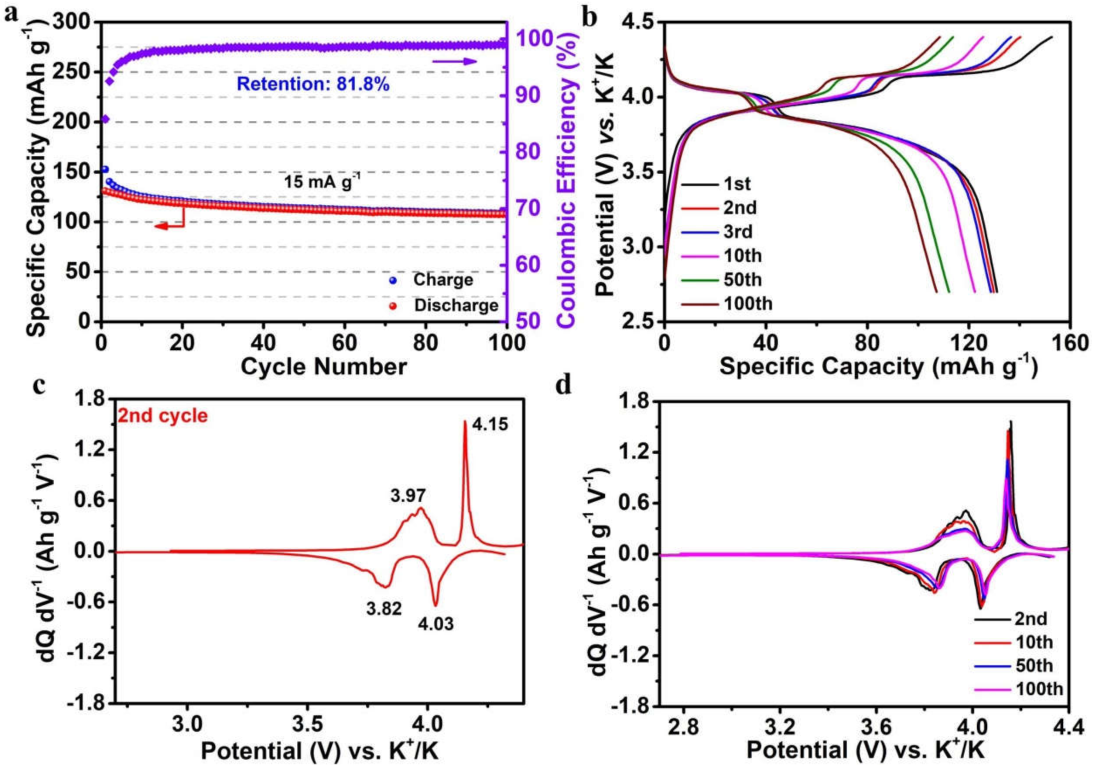

Supplementary Fig. 13 | Cycling performance (**a**) and voltage profiles (**b**) of the KMF-C|K metal cell at 15 mA g^{-1}. The corresponding differential capacity (dQ dV^{-1}) curves of the (**c**) 2nd cycle and (**d**) the 2nd, 10th, 50th, and 100th cycle.

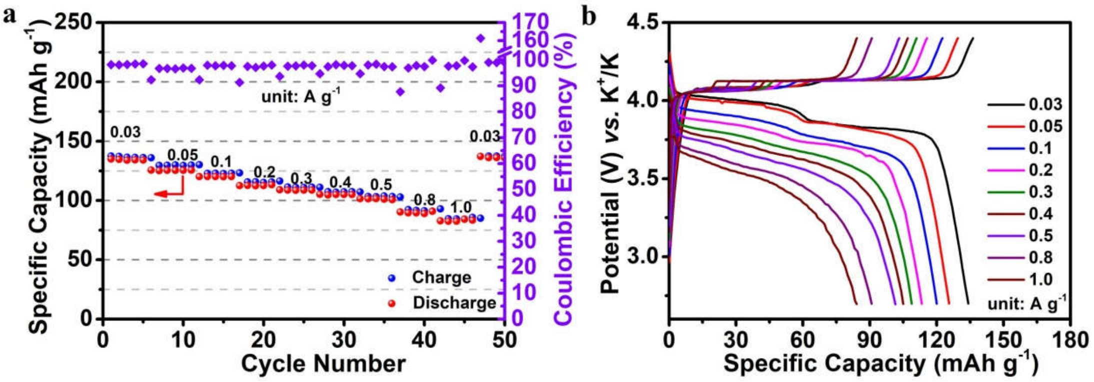

Supplementary Fig. 14 | Rate capability (**a**) and voltage profiles (**b**) of the KMF-EDTA|K metal cell under a constant charge specific current (30 mA g^{-1}) with different discharge specific currents from 0.03 to 1.0 A g^{-1}.

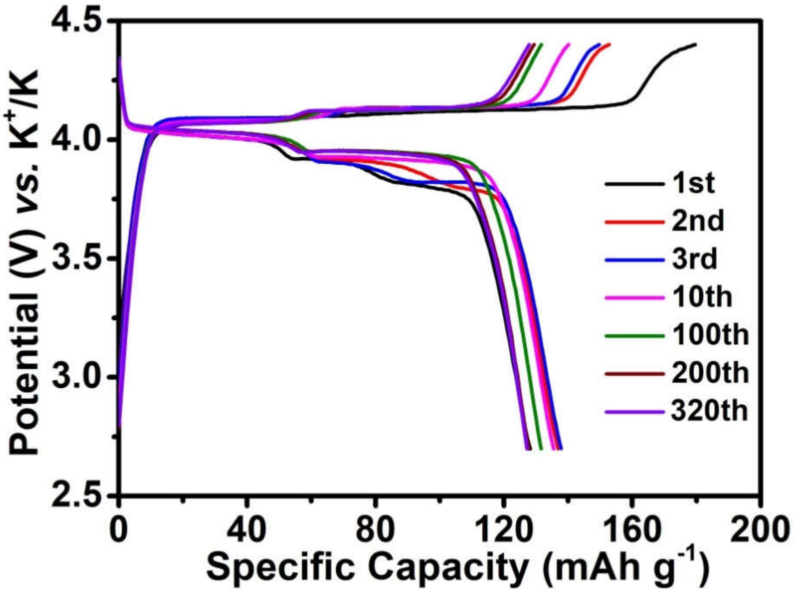

Supplementary Fig. 15 | Voltage profiles of the KMF-EDTA|K metal cell at 30 mA g^{-1}.

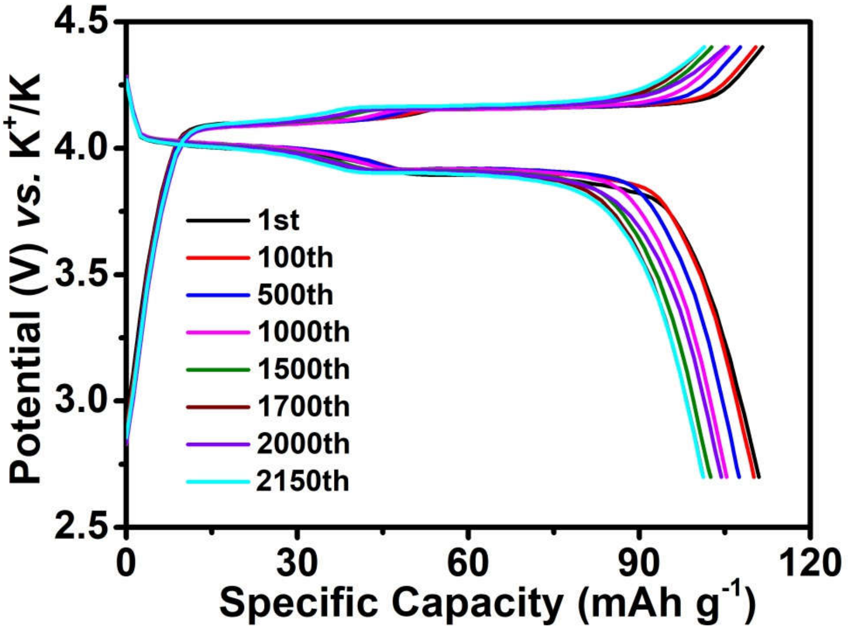

Supplementary Fig. 16 | Voltage profiles of the KMF-EDTA|K metal cell at 100 mA g^{-1}.

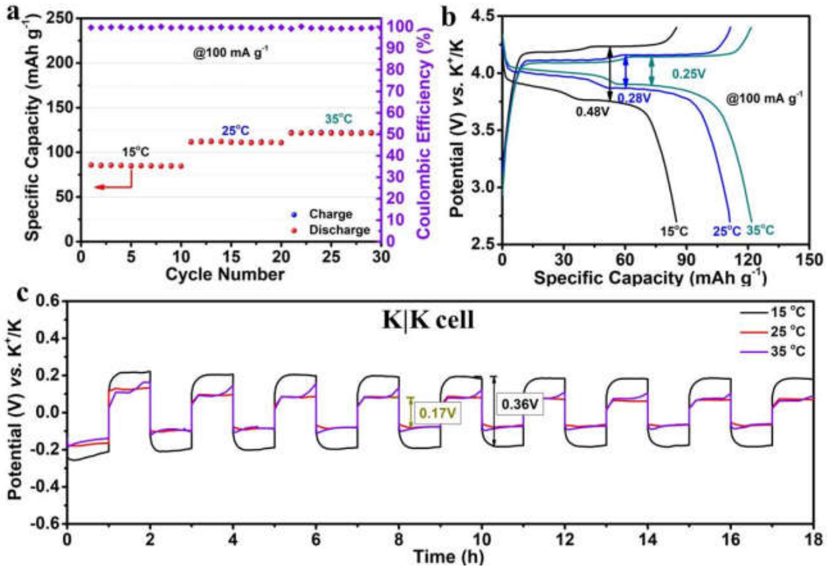

Supplementary Fig. 17 | Cycling performance of KMF-EDTA (**a**) and the corresponding voltage profiles under a specific current of 100 mA g^{-1} and different temperature (15, 25 and 35 ˚C) (**b**). **c** Voltage profiles of the symmetric K|K cells during continuous plating-stripping process under a specific current of 100 mA cm^{-1} and different temperature (15, 25 and 35 ˚C).

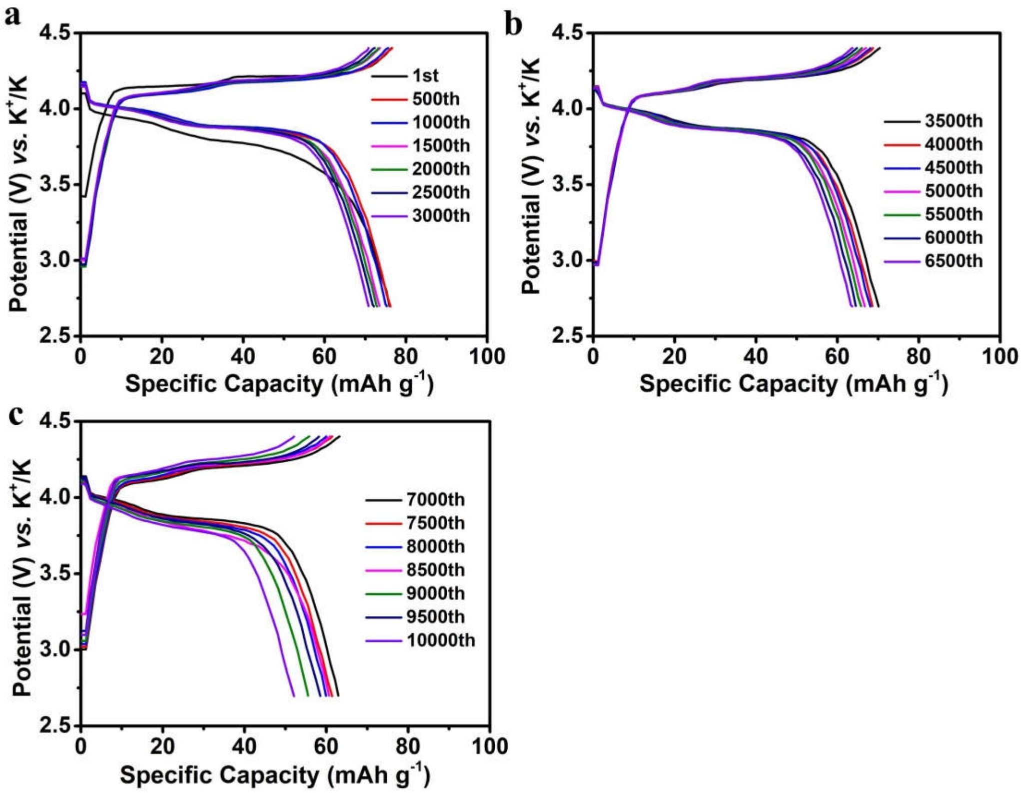

Supplementary Fig. 18 | a-c Voltage profiles of the KMF-EDTA|K metal cell for the different cycles at 500 mA g^{-1}.

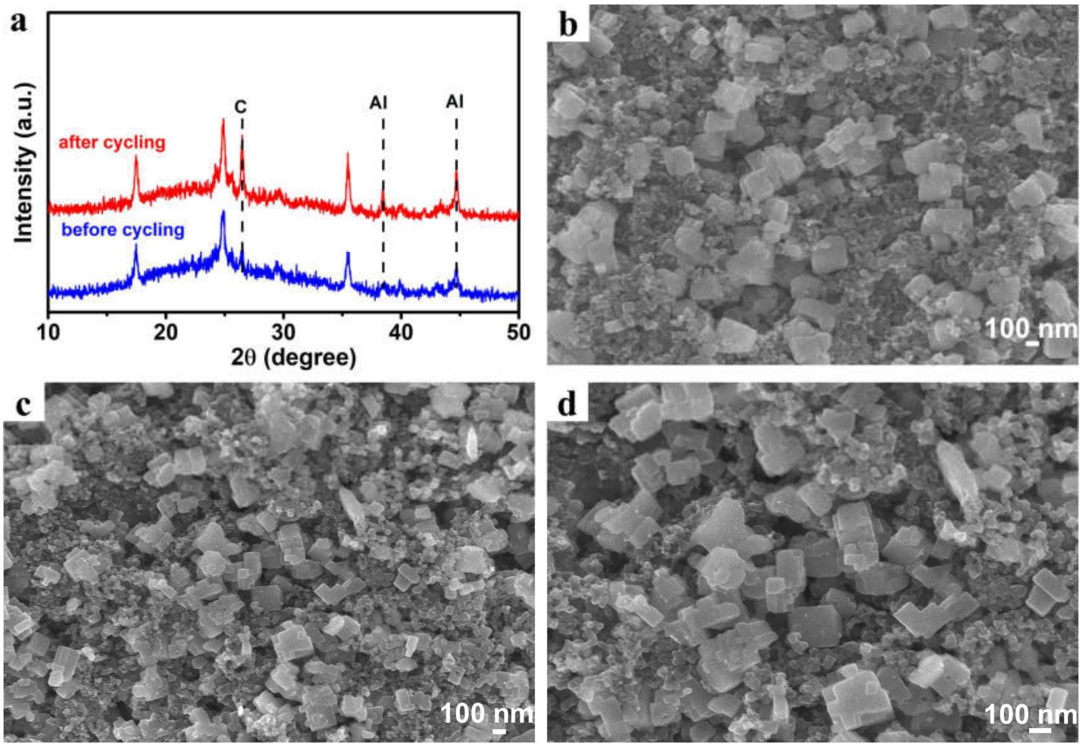

Supplementary Fig. 19 | a XRD patterns of the KMF-EDTA electrode before and after cycling for 1000 times at 100 mA g^{-1}. **b-d** SEM images of the (**b**) pristine KMF-EDTA electrode and (**c, d**) cycled KMF-EDTA electrode after 1000 cycles at 100 mA g^{-1}.

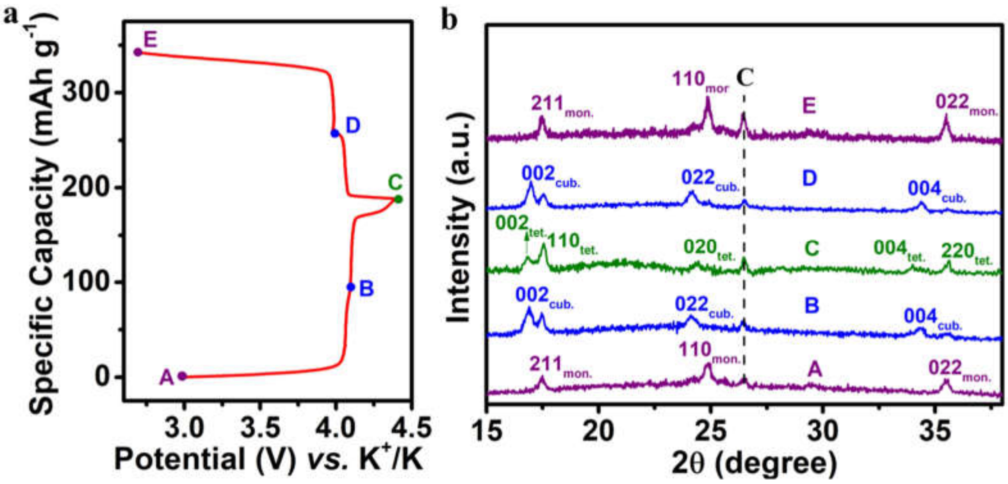

Supplementary Fig. 20 | The charge-discharge curves (**a**) and *ex situ* XRD patterns (**b**) of the KMF-EDTA electrode through the first cycle at 30 mA g^{-1}. The states tested by *ex situ* XRD are marked in (**a**) with points A-E.

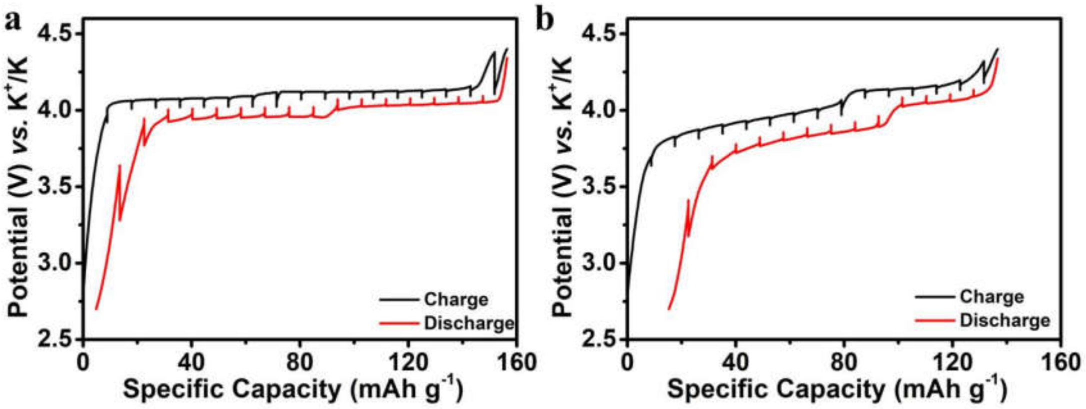

Supplementary Fig. 21 | Transient potential profiles versus specifc capacity obtained from GITT for K-ion insertion/extraction into/from the (**a**) KMF-EDTA and (**b**) KMF-C electrode.

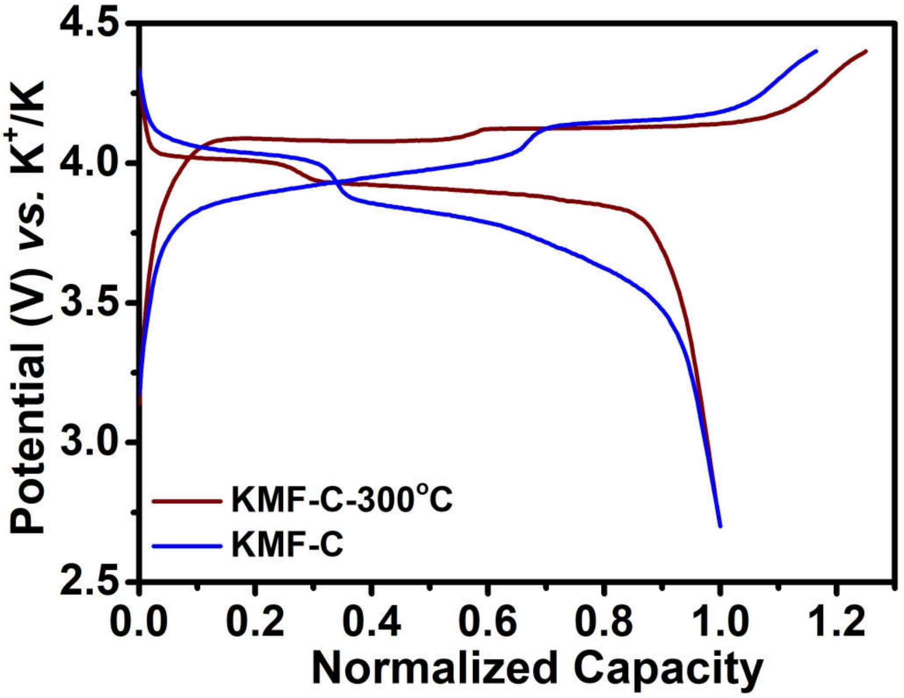

Supplementary Fig. 22 | Voltage profiles of the KMF-C and KMF-C-300˚C electrode which was treated at 300 ˚C for dehydrating completely.

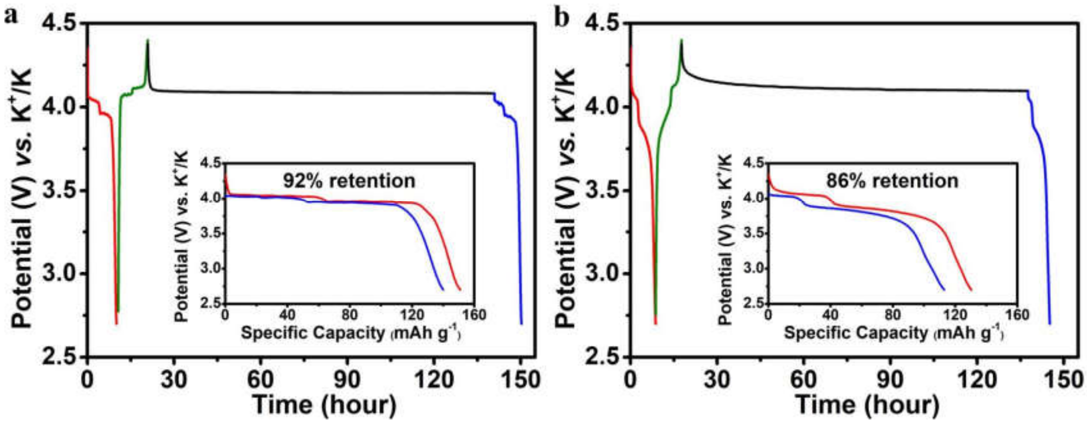

Supplementary Fig. 23 | The voltage-time curves of 120 h self-discharge process for the (**a**) KMF-EDTA|K and (**b**) KMF-C|K metal cell.

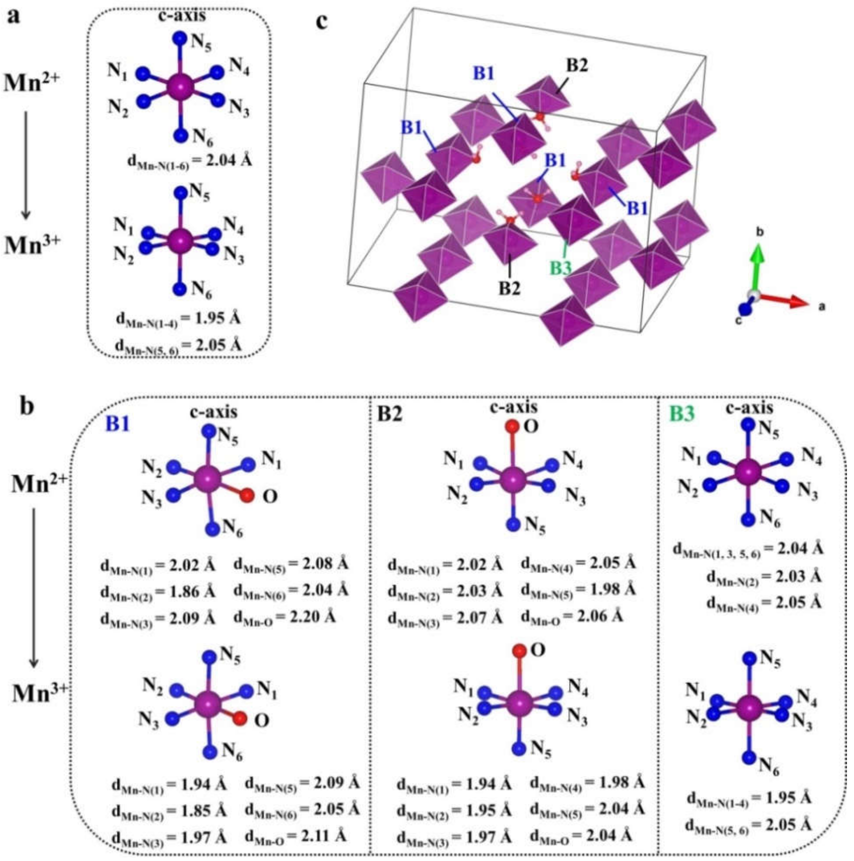

Supplementary Fig. 24 | Theoretical prediction of Mn-N bond changes during the second K-ion extraction in (**a**) defectless structure and (**b**) defective structure. **c** The schematic illustration of different MnN_6 octahedrons in the defective structure. The K atoms, C≡N bonds and FeC_6 octahedrons are omitted for clarity. The corresponding Mn-N bond length in the MnN_6 octahedrons marked with B1-B3 are shown in (**b**).

As shown in Supplementary Fig. 24a, for the defectless sample, the predicted Mn-N bond length exihibits -4.4% shrinkage for the four equatorial $Mn\text{-}N_{(1\text{-}4)}$ bonds and +0.049% elongation for the two axial $Mn\text{-}N_{(5\text{-}6)}$ upon the oxidation of Mn^{2+} to Mn^{3+}.

For the defective sample, the variation of the Mn-N bond length is highly uneven upon the oxidation of Mn^{2+} to Mn^{3+} (Supplementary Fig. 24b). Taking the B1 octahedron as an example, the variation of the Mn-N bond length is -4.0% for $Mn\text{-}N_1$, -0.054% for $Mn\text{-}N_2$, -5.7% for $Mn\text{-}N_3$, +0.48% for $Mn\text{-}N_5$, +0.49% for $Mn\text{-}N_6$. Such highly asymmetric Mn-N bond length variation may create strong local distortion and destroys the integrity of the defective KMF-C sample, contributing to the capacity decay upon cycling.

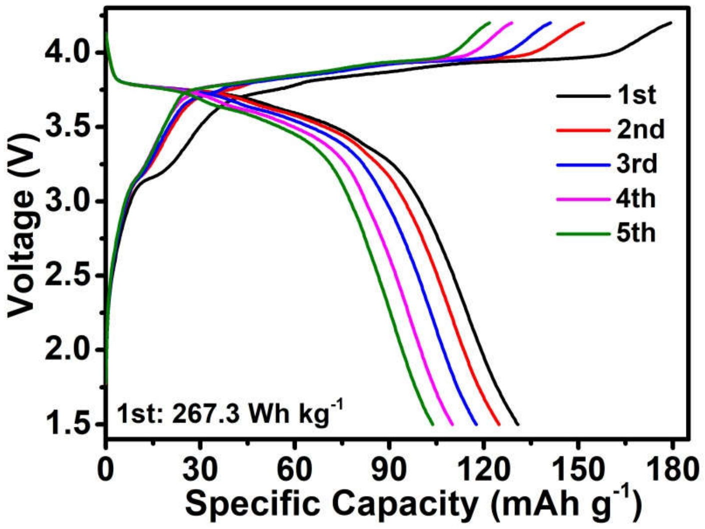

Supplementary Fig. 25 | Voltage profiles of the KMF-EDTA||Graphite full-cell at 15 mA g^{-1}. The specific energy of the 1st cycle was calculated based on the total mass of the cathode and anode materials.

Due to the poor initial Coulombic Efficiency (ICE) of the graphite anode (~65%, see Supplementary Fig. 26a) and the unoptimized full-cell parameters such as the N/P ratio (ratio of negative electrode capacity to positive electrode capacity), the KMF-EDTA||Graphite full-cell assembled by the KMF-EDTA cathode and the un-cycled graphite anode (the mass ratio of KMF-EDTA:Graphite in KMF-EDTA||Graphite full-cell is the same as that in KMF-EDTA||Cyc-graphite full-cell, *i.e.*, 1:0.6) can only show a discharge capacity of 130.8 mAh g^{-1} and a discharge specific energy of 267.3 Wh kg^{-1} (based on the total mass of the cathode and anode materials in the full-cell), much smaller than the values of the KMF-EDTA||Cyc-graphite full-cell in Fig. 5b (145 mAh g^{-1} and 331.5 Wh kg^{-1}).

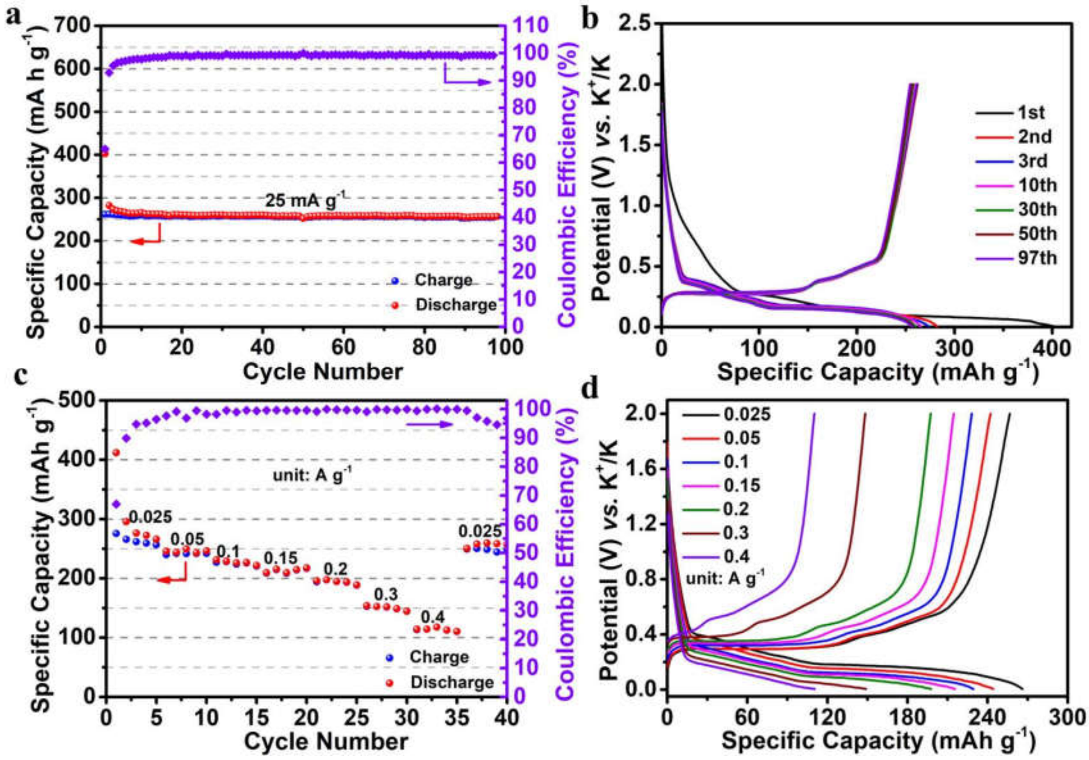

Supplementary Fig. 26 | Cycling performance (**a**) and the corresponding voltage profiles (**b**) of the graphite anode at 25 mA g^{-1}. Rate capability (**c**) and voltage profiles (**d**) of the graphite anode at different specific currents from 0.025 A g^{-1} to 0.4 A g^{-1}.

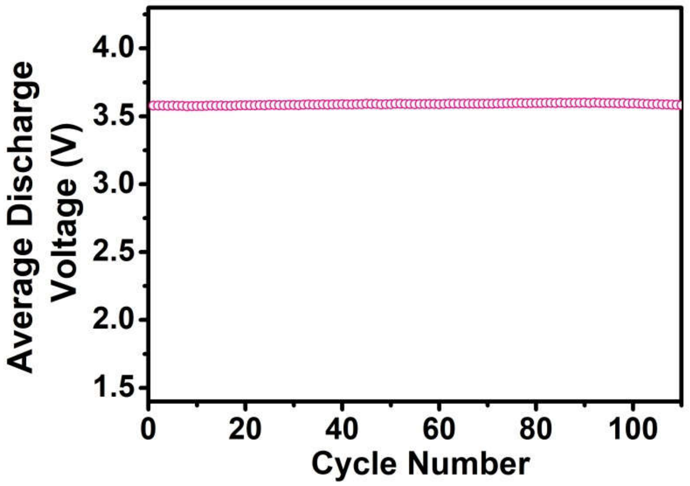

Supplementary Fig. 27 | Average discharge voltage of the KMF-EDTA||Cyc-graphite full-cell at 15 mA g^{-1}.

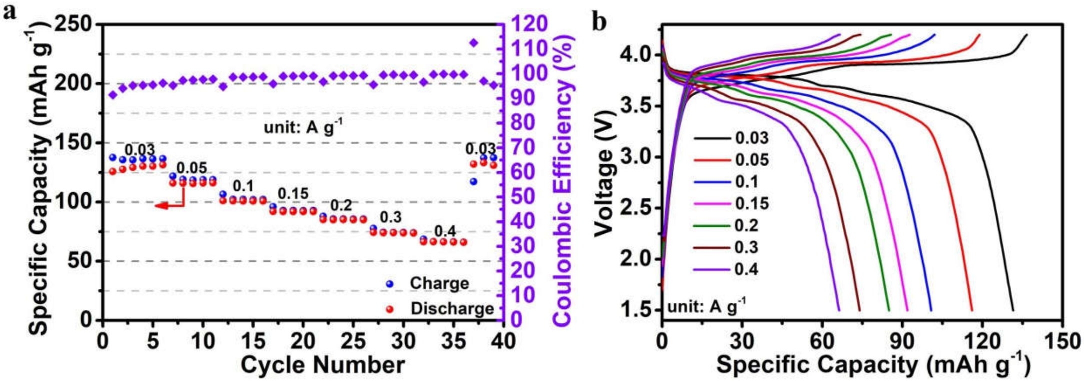

Supplementary Fig. 28 | Rate capability (**a**) and voltage profiles (**b**) of the KMF-EDTA||Cyc-graphite full-cell at different specific currents from 0.03 A g^{-1} to 0.4 A g^{-1}.

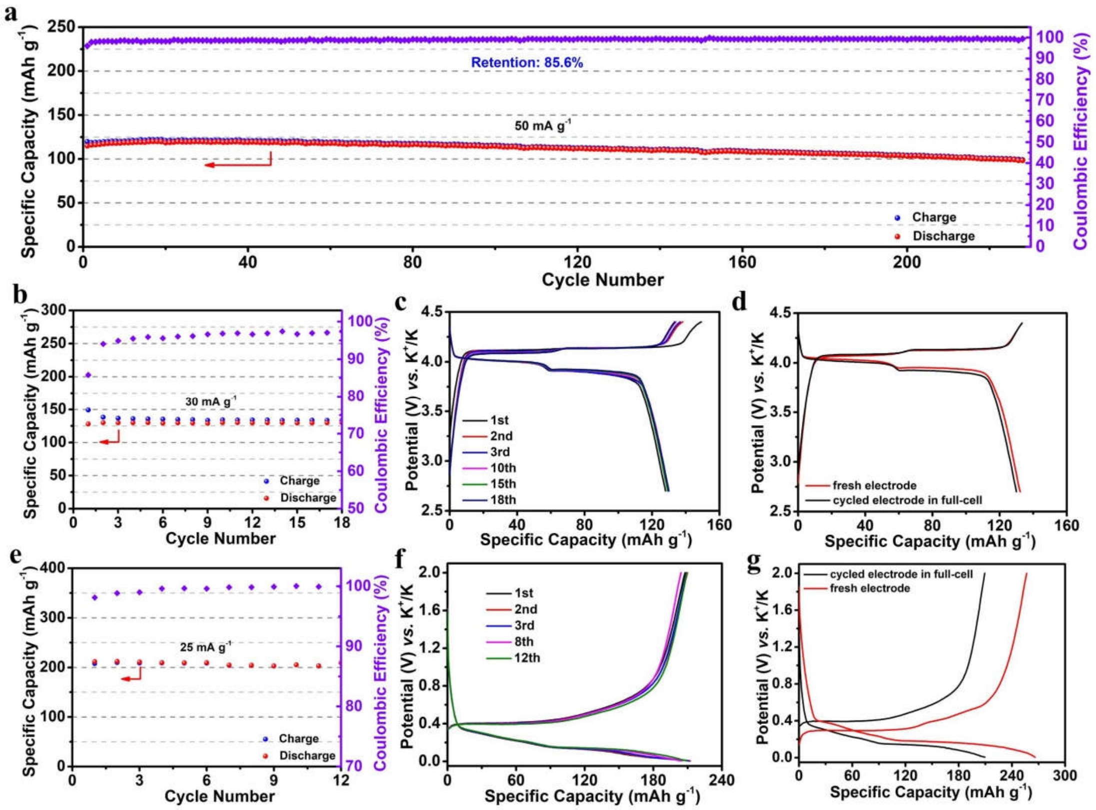

Supplementary Fig. 29 | a Cycling performance of the KMF-EDTA||Cyc-graphite full-cell at 50 mA g^{-1}. Cycling performance (**b,e**) and voltage profiles (**c,f**) for the cycled KMF-EDTA cathode (**b,c**) and cycled graphite anode (**e,f**) which were retrieved from the disassembled full-cell tested in (**a**). The comparison of the voltage curves for the fresh and cycled electrodes: (**d**) KMF-EDTA cathodes and (**g**) graphite anodes.

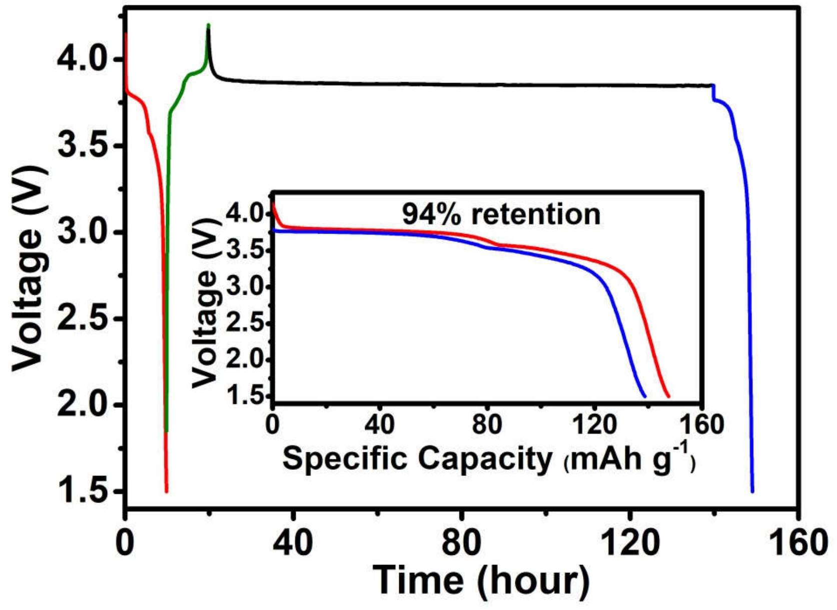

Supplementary Fig. 30 | The voltage-time curve of 120 h self-discharge process for the KMF-EDTA||Cyc-graphite full-cell.

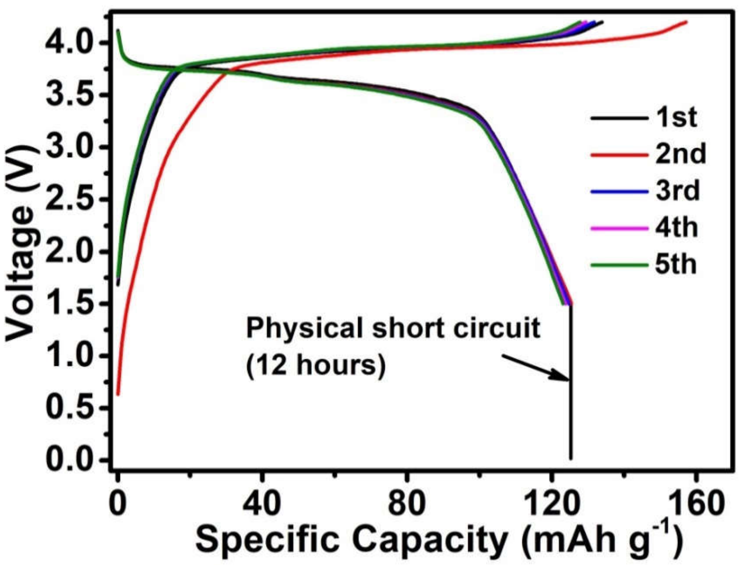

Supplementary Fig. 31 | Zero voltage storage. The voltage profiles for the KMF-EDTA||Cyc-graphite full-cell at 30 mA g^{-1} which was physically short-circuited at 0 V for 12 h at the end of 1st discharge process.

Supplementary Table 1 | Structural parameters of the KMF-C sample obtained from Rietveld analysis.

Atom	Wyckoff	x	y	z	Occupancy
Fe	2a	0	0	0	1
Mn	2a	0	0.5	0.5	1
C1	4e	0.56939	0.65326	0.25133	1
C2	4e	0.96034	0.78003	0.15881	1
C3	4e	0.69214	0.51137	0.55103	1
N1	4e	0.46115	0.24490	0.83820	1
N2	4e	0.03557	0.34893	0.73401	1
N3	4e	0.20263	0.47542	0.39991	1
K	4e	0.79647	0.94101	0.47822	0.9
S.G. P21/n a = 10.14201, b = 7.25722, c = 7.03956 Å, α=γ=90° β=90.52128°					
R_p= 3.82%, R_{wp}= 4.83%, R_{exp}=4.30%					

Supplementary Table 2 | ICP-MS results for the KMF-C and KMF-EDTA samples.

Sample	K:Mn:Fe	molecular formula*
KMF-C	1.72:1:0.92	$K_{1.72}Mn[Fe(CN)_6]_{0.92}\square_{0.08}\bullet 1.43H_2O$
KMF-EDTA	1.94:1:0.992	$K_{1.94}Mn[Fe(CN)_6]_{0.994}\square_{0.006}\bullet 0.08H_2O$**

*The molecular formula of the samples was calculated based on the ICP-MS and TGA results of the KMF-C and KMF-EDTA samples.

**The uncertainties for the measured molar ratio of K and Fe versus Mn are 0.5% and 0.1%, respectively.

Supplementary Table 3 | Structural parameters of the KMF-EDTA sample obtained from Rietveld analysis.

Atom	Wyckoff.	x	y	z	Occupancy
Fe	2a	0	0	0	1
Mn	2a	0	0.5	0.5	1
C1	4e	0.58918	0.66858	0.19488	1
C2	4e	0.95256	0.73440	0.16922	1
C3	4e	0.70486	0.52585	0.57268	1
N1	4e	0.46562	0.23612	0.83789	1
N2	4e	0.03153	0.35104	0.74134	1
N3	4e	0.20334	0.47264	0.39687	1
K	4e	0.751650	0.93746	0.47994	0.97
S.G. $P2_1/n$ a = 10.0912(3), b = 7.3243(2), c = 6.9442(2) Å, α=γ=90° β=90.016(6)°					
R_p= 1.14%, R_{wp}= 1.48%, R_{exp}=1.21%, Chi2=1.50					

Supplementary Table 4 | Comparison of K content, water content, and $[Fe(CN)_6]$ vacancy of some reported KMF.

Sample	Chemical formula	K content	Water content (wt%)	$[Fe(CN)_6]^{4-}$ vacancy (*vs.* Mn)
KMF-EDTA	$K_{1.94}Mn[Fe(CN)_6]_{0.994}$•$0.08H_2O$ (This work)	1.94	0.44	0.006
KMF-C	$K_{1.72}Mn[Fe(CN)_6]_{0.92}$•$1.43H_2O$ (This work)	1.72	7.51	0.08
KMF-1	$K_{1.89}Mn[Fe(CN)_6]_{0.92}$•$0.75H_2O$[10]	1.89	4.0	0.08
KMF-2	$K_{1.75}Mn[Fe(CN)_6]_{0.93}$•$0.16H_2O$[14]	1.75	0.9	0.07
KMF-3	$K_{1.85}Mn[Fe(CN)_6]_{0.98}$•$0.7H_2O$[15]	1.85	3.5	0.02
KMF-4	$K_{1.80}Na_{0.05}Mn[Fe(CN)_6]_{0.94}$•$0.49H_2O$[16]	1.80	4.7	0.06
KMF-5	$K_{1.84}Mn[Fe(CN)_6]_{0.84}$•$1.41H_2O$[17]	1.84	7.7	0.13
KMF-6	$K_{1.67}Mn[Fe(CN)_6]_{0.91}$•$0.73H_2O$[18]	1.67	4.05	0.09
KMF-Citrate	$K_{1.91}Mn[Fe(CN)_6]_{0.97}$•$0.43H_2O$[19]	1.91	2.25	0.03

Supplementary Table 5 | Average discharge voltage and specific energy versus specific capacity of some reported cathodes for LIBs, NIBs, and KIBs.

Cathodes	Voltage window (V *vs.* A^+/A)	Average discharge potential (V *vs.* A^+/A)	Discharge capacity (mAh g^{-1})	Specific energy (Wh kg^{-1})
Potassium ion batteries (KIBs)				
KMF-EDTA (This work)	2.7-4.4	3.941	154.7	609.7
$K_{1.75}Mn[Fe(CN)_6]_{0.93}$• $0.16H_2O$[14]	2.0-4.5	3.8	141	535.8
$K_{1.91}Mn[Fe(CN)_6]_{0.97}$• $0.43H_2O$[19]	3.5-4.3	~4.0	119	~476
$K_{0.51}V_2O_5$[20]	2.0-4.5	3.03	131	396.9
$K_{0.5}MnO_2$[1]	2-4.2	2.96	126.8	375.3
$KVPO_4F$[21]	3-5.0	4.3	105	451.5
$KVOPO_4$[22]	2.0-4.6	3.65	115	419.8
$K_3V_2(PO_4)_2F_3$[23]	2.0-4.6	3.7	104	384.8
Sodium ion batteries (NIBs)				
$Na_2MnFe(CN)_6$[24]	2-3.8	3.39	160	542
P2-$Na_{0.67}Fe_{0.2}Cu_{0.14}Mn_{0.66}O_2$[25]	1.5-4.3	2.71	203	548.1
P2-$Na_{0.7}Fe_{0.5}Mn_{0.5}O_2$[25]	1.5-4.3	2.7	195	526.5
P2-$Na_{0.67}Mg_{0.3}Mn_{0.7}O_2$[25]	1.5-4.5	2.47	205	506.35
O3-$NaNi_{0.5}Mn_{0.3}Ti_{0.2}O_2$[25]	2.2-4	3.07	150	460.5
$Na_3V_2(PO_4)_2F_3$[25]	2-4.3	3.64	122.4	445.5
Lithium ion batteries (LIBs)				
$LiFePO_4$[26]	2.5-3.8	3.4	170	578
$LiCoO_2$[27]	2.7-4.3	3.6	140	504
$LiMn_2O_4$[28]	3.6-4.5	4.1	121	496.1

Supplementary Table 6 | The valence state of K ion in the transition state for the KMF-EDTA system and KMF-C system. (The locations of K_1-K_{31} in the table are shown in fig. a and b for KMF-EDTA and KMF-C, respectively.)

No. of K ion	KMF-EDTA	KMF-C	No. of K ion	KMF-EDTA	KMF-C
K_1	0.801	0.762	K_{17}	0.908	0.908
K_2	0.910	0.865	K_{18}	0.908	0.908
K_3	0.909	0.841	K_{19}	0.908	0.908
K_4	0.909	0.830	K_{20}	0.909	0.909
K_5	0.909	0.883	K_{21}	0.908	0.908
K_6	0.908	0.836	K_{22}	0.909	0.909
K_7	0.908	0.861	K_{23}	0.908	0.908
K_8	0.908	0.908	K_{24}	0.908	0.908
K_9	0.908	0.908	K_{25}	0.908	0.908
K_{10}	0.908	0.908	K_{26}	0.910	0.909
K_{11}	0.908	0.908	K_{27}	0.909	0.909
K_{12}	0.908	0.908	K_{28}	0.908	0.908
K_{13}	0.908	0.908	K_{29}	0.908	0.907
K_{14}	0.909	0.909	K_{30}	0.908	0.908
K_{15}	0.909	0.909	K_{31}	0.908	0.908
K_{16}	0.908	0.908			

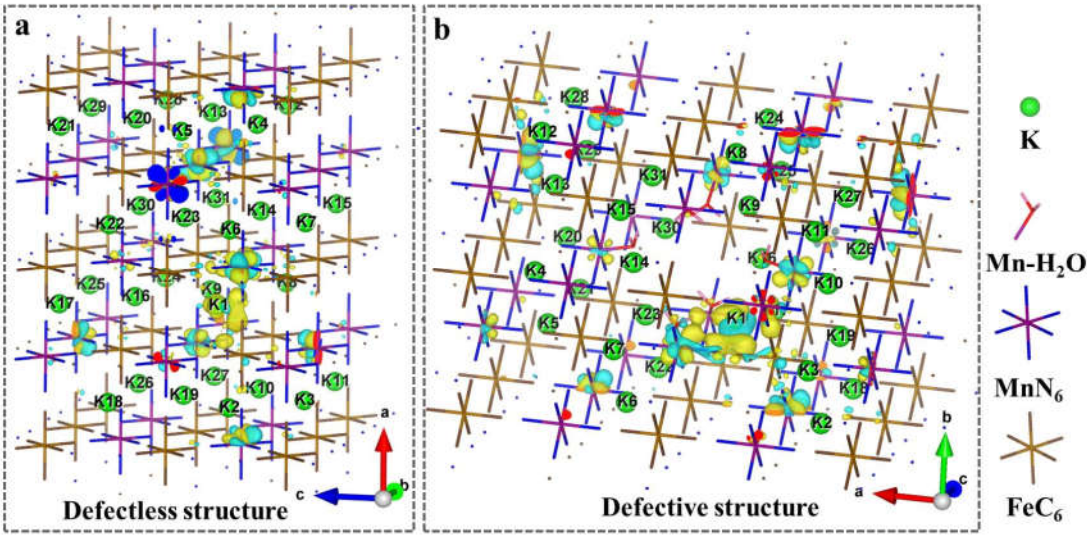

Supplementary Table 6 shows the calculated valence state of K ion in the transition state for KMF-EDTA system (shown in Fig. 4d) and KMF-C system (shown in Fig. 4e). Supplementary fig. a and b following Supplementary Table 6 depict the locations of K_1-K_{31} listed in the table. Among them, K_1 ion is the diffusion ion which participates in the CI-NEB process. K_2, K_3, K_4, K_5, K_6 and K_7 are the host K atoms which neighboring the water molecules and vacancies in the KMF-C structure. It can be seen that when the K atom is far away from the influence of water molecules and vacancies (K_8 ~ K_{31} in KMF-EDTA and KMF-C), its valence is closer to the intrinsic valence of +1. However, compare with KMF-EDTA system, the valence states of K_1 ~ K_7 atoms in KMF-C system deviates fiercely from their intrinsic valence state, which leads a relatively higher energy of KMF-C. Fig. 4d and 4e shows that due to the presence of water molecules and vacancies, the water molecules exert coulombic attraction with the diffused K_1 ion. Thus, the electron cloud rearrangement occurs around the diffused K ion and water molecules which leads difficulty in diffusion of the K ion and results in a high diffusion barrier. Therefore, due to the influence of water molecules and vacancies on the charge of K ion, the KMF-C system has a higher K ion diffusion barrier, which results in large voltage polarization during charge-discharge.

Supplementary Table 7 | The ICP-MS results of the dissolved Fe and Mn ions in the electrolyte for the KMF-C and KMF-EDTA electrode after 100 cycles

	Fe (mg L^{-1})	Mn (mg L^{-1})
KMF-C	8	11
KMF-EDTA	2	4

Supplementary Table 8 | Specific energy of some reported K-ion full-cells.

Positive|negative materials	The mass ratio of cathode material:anode material	Average discharge voltage (V)	Discharge capacity (mAh g^{-1})*	Specific energy (Wh kg^{-1})*
$K_{1.94}Mn[Fe(CN)_6]_{0.994}$•0.08 H_2O|Graphite (This work)	1:0.6	3.58	92.6	331.5
$K_{1.75}Mn[Fe(CN)6]_{0.93}$•0.16 H_2O|Graphite[14]	1:0.6	3.5	68.8	240.8
$K_{0.61}Fe[Fe(CN)_6]_{0.92}$•0.32$H_2O$|Graphite[29]	1:1	2.9	40	116
$K_{0.22}Fe[Fe(CN)_6]_{0.805}$•4.01 H_2O|Super P[30]	1.1:1	2.2	34.8	76.6
$K_{1.92}Fe[Fe(CN)6]_{0.94}$•0.5$H_2O$|$K_2$TP[31]	1:0.6	2.8	68.8	192.6
$K_3V_2(PO_4)_2F_3$|Graphite[23]	1:0.48	3.4	56.8	193.1
$K_2C_6O_6$|$K_4C_6O_6$[32]	1:1.2	1.1	31.8	35
$K_{0.5}MnO_2$|Graphite[1]	2:1	2.64	73.7	194.6
$K_{0.3}MnO_2$|Hard carbon/carbon black[33]	1.35:1	2.05	51.7	106
$K_{0.7}Fe_{0.5}Mn_{0.5}O_2$|Soft carbon[34]	2.1:1	1.6	55.5	88.8
$K_{0.6}CoO_2$|hard carbon[35]	3:1	1.9	54	102.6
$KCrO_2$|Graphite[36]	3:1	1.9	67.5	128.3
$K_{0.6}CoO_2$|Graphite[37]	2:1	2.5	35.3	88.3
$K_{0.65}Fe_{0.5}Mn_{0.5}O_2$|Hard carbon[38]	2.1:1	2	51.4	102.8
$K_{0.77}MnO_2$•0.23H_2O|Hard-	1.8:1	1.8	77	138.6

soft composite carbon[39]				
$K_{0.8}CrO_2$|Graphite[40]	1:1.4	2.2	33	72.6
$K_{0.51}V_2O_5$|Graphite[20]	1:1	2.53	47	119

*Discharge capacity and specific energy is calculated based on the total mass of the cathode and anode materials in the full-cell.

Supplementary Table 9 | Comparison of the K-ion lab-scale cell reported in the present work with other electrochemical energy storage systems for grid-scale applications.

	KMF-EDTA‖Graphite (This work)	Lead-acid battery[41]	Flow battery (Vanadium redox battery)[41,42]	Ni-MH battery[43]	Na-S battery[41]	Super-capacitor[44]	Li-ion battery ($LiFePO_4$‖Graphite)[41,45]
Specific energy (Wh kg^{-1})	331.5/2= 165.75*	25-40	10-25	50-85	120-150	5-10	160
Average voltage	3.58 V	2.1 V	1.4 V	1.2 V	2.1 V	2.0 V	3.2 V
Self-discharge rate	Medium	Medium	Low	Medium	Low	Medium	Low
Cost	Good	Excellent	Medium	Medium	Good	Poor	Medium
Abundance of key element in the Earth's crust (ppm)	Fe (50500)<br>Mn (950)<br>K (25800)<br>P (1000)	Pb (14)	V (120)	Ni (84)<br>La (34)	Na (27500)<br>S (520)	C	Li (20)

*The specific energy value of the KMF‖Graphite battery is arbitrarily estimated by halving the corresponding lab-scale cell value (originally calculated based on the total mass of cathode and anode materials), as reported in Supplementary Reference 13.

Supplementary References:

1. Deng, L. *et al.* A Nonflammable Electrolyte Enabled High Performance $K_{0.5}$ MnO_2 Cathode for Low-Cost Potassium-Ion Batteries. *ACS Energy Lett.* **5**, 1916–1922 (2020).
2. Kresse, G. & Furthmüller, J. Efficient iterative schemes for ab initio total-energy calculations using a plane-wave basis set. *Phys. Rev. B* **54**, 11169–11186 (1996).
3. Kresse, G. & Furthmüller, J. Efficiency of ab-initio total energy calculations for metals and semiconductors using a plane-wave basis set. *Comput. Mater. Sci.* **6**, 15–50 (1996).
4. Kresse, G. & Joubert, D. From ultrasoft pseudopotentials to the projector augmented-wave method. *Phys. Rev. B* **59**, 1758–1775 (1999).
5. Blöchl, P. E. Projector augmented-wave method. *Phys. Rev. B* **50**, 17953–17979 (1994).
6. Henkelman, G., Uberuaga, B. P. & Jónsson, H. A climbing image nudged elastic band method for finding saddle points and minimum energy paths. *J. Chem. Phys.* **113**, 9901–9904 (2000).
7. Chen, H. *et al.* Defect-Enhanced CO_2 Reduction Catalytic Performance in O-Terminated MXenes. *ChemSusChem* **13**, 5690–5698 (2020).
8. Courtney, I. A., Tse, J. S., Mao, O., Hafner, J. & Dahn, J. R. Ab initio calculation of the lithium-tin voltage profile. *Phys. Rev. B* **58**, 15583–15588 (1998).
9. Westre, T. E. *et al.* A Multiplet Analysis of Fe K-Edge 1*s* → 3*d* Pre-Edge Features of Iron Complexes. *J. Am. Chem. Soc.* **119**, 6297–6314 (1997).
10. Xue, L. *et al.* Low-Cost High-Energy Potassium Cathode. *J. Am. Chem. Soc.* **139**, 2164–2167 (2017).
11. Mullaliu, A., Asenbauer, J., Aquilanti, G., Passerini, S. & Giorgetti, M. Highlighting the Reversible Manganese Electroactivity in Na-Rich Manganese Hexacyanoferrate Material for Li- and Na-Ion Storage. *Small Methods* **4**,

1900529 (2020).

12. Giorgetti, M., Aquilanti, G., Ciabocco, M. & Berrettoni, M. Anatase-driven charge transfer involving a spin transition in cobalt iron cyanide nanostructures. *Phys. Chem. Chem. Phys.* **17**, 22519–22522 (2015).
13. Jiang, L. *et al.* Building aqueous K-ion batteries for energy storage. *Nat. Energy* **4**, 495–503 (2019).
14. Bie, X., Kubota, K., Hosaka, T., Chihara, K. & Komaba, S. A novel K-ion battery: hexacyanoferrate (II)/graphite cell. *J. Mater. Chem. A* **5**, 4325–4330 (2017).
15. Sun, Y. *et al.* Potassium manganese hexacyanoferrate/graphene as a high-performance cathode for potassium-ion batteries. *New J. Chem.* **43**, 11618–11625 (2019).
16. Zhou, A. *et al.* Size-, Water-, and Defect-Regulated Potassium Manganese Hexacyanoferrate with Superior Cycling Stability and Rate Capability for Low-Cost Sodium-Ion Batteries. *Small* **15**, 1902420 (2019).
17. Jiang, X., Liu, H., Song, J., Yin, C. & Xu, H. Hierarchical mesoporous octahedral $K_2Mn_{1-x}Co_xFe(CN)_6$ as a superior cathode material for sodium-ion batteries. *J. Mater. Chem. A* **4**, 16205–16212 (2016).
18. Liu, Y., He, D., Han, R., Wei, G. & Qiao, Y. Nanostructured potassium and sodium ion incorporated Prussian blue frameworks as cathode materials for sodium-ion batteries. *Chem. Commun.* **53**, 5569–5572 (2017).
19. Fiore, M. *et al.* Paving the Way toward Highly Efficient, High-Energy Potassium-Ion Batteries with Ionic Liquid Electrolytes. *Chem. Mater.* **32**, 7653–7661 (2020).
20. Zhu, Y.-H. *et al.* Reconstructed Orthorhombic V_2O_5 Polyhedra for Fast Ion Diffusion in K-Ion Batteries. *Chem* **5**, 168–179 (2019).
21. Kim, H. *et al.* A New Strategy for High-Voltage Cathodes for K-Ion Batteries: Stoichiometric $KVPO_4F$. *Adv. Energy Mater.* **8**, 1801591 (2018).
22. Liao, J. *et al.* Competing with other polyanionic cathode materials for potassium-ion batteries via fine structure design: new layered $KVOPO_4$ with a

tailored particle morphology. *J. Mater. Chem. A* **7**, 15244–15251 (2019).

23. Lin, X., Huang, J., Tan, H., Huang, J. & Zhang, B. $K_3V_2(PO_4)_2F_3$ as a robust cathode for potassium-ion batteries. *Energy Storage Mater.* **16**, 97–101 (2019).

24. Bauer, A. *et al.* The Scale-up and Commercialization of Nonaqueous Na-Ion Battery Technologies. *Adv. Energy Mater.* **8**, 1702869 (2018).

25. Mariyappan, S., Wang, Q. & Tarascon, J. M. Will Sodium Layered Oxides Ever Be Competitive for Sodium Ion Battery Applications? *J. Electrochem. Soc.* **165**, A3714–A3722 (2018).

26. Choi, D. & Kumta, P. N. Surfactant based sol–gel approach to nanostructured $LiFePO_4$ for high rate Li-ion batteries. *J. Power Sources* **163**, 1064–1069 (2007).

27. Tarascon, J.-M. & Armand, M. Issues and challenges facing rechargeable lithium batteries. *Nature* **414**, 359–367 (2001).

28. Li, W.-J. *et al.* Multifunctional conducing polymer coated $Na_{1+x}MnFe(CN)_6$ cathode for sodium-ion batteries with superior performance via a facile and one-step chemistry approach. *Nano Energy* **13**, 200–207 (2015).

29. Zhu, Y.-H. *et al.* High-Energy-Density Flexible Potassium-Ion Battery Based on Patterned Electrodes. *Joule* **2**, 736–746 (2018).

30. Zhang, C. *et al.* Potassium Prussian Blue Nanoparticles: A Low-Cost Cathode Material for Potassium-Ion Batteries. *Adv. Funct. Mater.* **27**, 1604307 (2017).

31. Liao, J. *et al.* A potassium-rich iron hexacyanoferrate/dipotassium terephthalate@carbon nanotube composite used for K-ion full-cells with an optimized electrolyte. *J. Mater. Chem. A* **5**, 19017–19024 (2017).

32. Zhao, Q. *et al.* Oxocarbon Salts for Fast Rechargeable Batteries. *Angew. Chemie* **128**, 12716–12720 (2016).

33. Vaalma, C., Giffin, G. A., Buchholz, D. & Passerini, S. Non-Aqueous K-Ion Battery Based on Layered $K_{0.3}MnO_2$ and Hard Carbon/Carbon Black. *J. Electrochem. Soc.* **163**, A1295–A1299 (2016).

34. Wang, X. *et al.* Earth Abundant Fe/Mn-Based Layered Oxide Interconnected Nanowires for Advanced K-Ion Full Batteries. *Nano Lett.* **17**, 544–550 (2017).

35. Deng, T. *et al.* Self-Templated Formation of P2-type $K_{0.6}CoO_2$ Microspheres for High Reversible Potassium-Ion Batteries. *Nano Lett.* **18**, 1522–1529 (2018).
36. Kim, H. *et al.* Stoichiometric Layered Potassium Transition Metal Oxide for Rechargeable Potassium Batteries. *Chem. Mater.* **30**, 6532–6539 (2018).
37. Kim, H. *et al.* K-Ion Batteries Based on a P2-Type $K_{0.6}CoO_2$ Cathode. *Adv. Energy Mater.* **7**, 1700098 (2017).
38. Deng, T. *et al.* Layered P2-Type $K_{0.65}Fe_{0.5}Mn_{0.5}O_2$ Microspheres as Superior Cathode for High-Energy Potassium-Ion Batteries. *Adv. Funct. Mater.* **28**, 1800219 (2018).
39. Lin, B. *et al.* Birnessite Nanosheet Arrays with High K Content as a High-Capacity and Ultrastable Cathode for K-Ion Batteries. *Adv. Mater.* **31**, 1900060 (2019).
40. Naveen, N. *et al.* Highly stable P′3-$K_{0.8}CrO_2$ cathode with limited dimensional changes for potassium ion batteries. *J. Power Sources* **430**, 137–144 (2019).
41. Yang, Z. *et al.* Electrochemical Energy Storage for Green Grid. *Chem. Rev.* **111**, 3577–3613 (2011).
42. Rydh, C. J. Environmental assessment of vanadium redox and lead-acid batteries for stationary energy storage. *J. Power Sources* **80**, 21–29 (1999).
43. Gifford, P., Adams, J., Corrigan, D. & Venkatesan, S. Development of advanced nickel/metal hydride batteries for electric and hybrid vehicles. *J. Power Sources* **80**, 157–163 (1999).
44. Opiyo, N. Energy storage systems for PV-based communal grids. *J. Energy Storage* **7**, 1–12 (2016).
45. Hesse, H., Schimpe, M., Kucevic, D. & Jossen, A. Lithium-Ion Battery Storage for the Grid—A Review of Stationary Battery Storage System Design Tailored for Applications in Modern Power Grids. *Energies* **10**, 2107 (2017).
